# Multi-center validation of machine learning model for preoperative prediction of postoperative mortality

**DOI:** 10.1038/s41746-022-00625-6

**Published:** 2022-07-12

**Authors:** Seung Wook Lee, Hyung-Chul Lee, Jungyo Suh, Kyung Hyun Lee, Heonyi Lee, Suryang Seo, Tae Kyong Kim, Sang-Wook Lee, Yi-Jun Kim

**Affiliations:** 1 School of Medicine, Kyungpook National University, Daegu, Republic of Korea; 2 Department of Anesthesiology and Pain Medicine, Seoul National University Hospital, Seoul National University College of Medicine, Seoul, Republic of Korea; 3 Department of Urology, Asan Medical Center, University of Ulsan College of Medicine, Seoul, Republic of Korea; 4 Department of Digital Health, SAIHST, Sungkyunkwan University, Seoul, Republic of Korea; 5 Bioinformatics Collaboration Unit, Department of Biomedical Systems informatics, Yonsei University College of medicine, Seoul, Republic of Korea; 6 Department of Nursing, SMG-SNU Boramae Medical Center, Seoul National University College of Medicine, Seoul, South Korea; 7 Department of Anesthesiology and Pain Medicine, SMG-SNU Boramae Medical Center, Seoul National University College of Medicine, Seoul, South Korea; 8 Department of Anesthesiology and Pain Medicine, Asan Medical Center, University of Ulsan College of Medicine, Seoul, Republic of Korea; 9 Institute of Convergence Medicine, Ewha Womans University Mokdong Hospital, Seoul, Republic of Korea

**Keywords:** Outcomes research, Health policy

## Abstract

Accurate prediction of postoperative mortality is important for not only successful postoperative patient care but also for information-based shared decision-making with patients and efficient allocation of medical resources. This study aimed to create a machine-learning prediction model for 30-day mortality after a non-cardiac surgery that adapts to the manageable amount of clinical information as input features and is validated against multi-centered rather than single-centered data. Data were collected from 454,404 patients over 18 years of age who underwent non-cardiac surgeries from four independent institutions. We performed a retrospective analysis of the retrieved data. Only 12–18 clinical variables were used for model training. Logistic regression, random forest classifier, extreme gradient boosting (XGBoost), and deep neural network methods were applied to compare the prediction performances. To reduce overfitting and create a robust model, bootstrapping and grid search with tenfold cross-validation were performed. The XGBoost method in Seoul National University Hospital (SNUH) data delivers the best performance in terms of the area under receiver operating characteristic curve (AUROC) (0.9376) and the area under the precision-recall curve (0.1593). The predictive performance was the best when the SNUH model was validated with Ewha Womans University Medical Center data (AUROC, 0.941). Preoperative albumin, prothrombin time, and age were the most important features in the model for each hospital. It is possible to create a robust artificial intelligence prediction model applicable to multiple institutions through a light predictive model using only minimal preoperative information that can be automatically extracted from each hospital.

## Introduction

Approximately 250 million surgeries are performed worldwide every year^1^. As the number of surgical procedures increases every year, postoperative complications also increase^2^. A postoperative complication is a major concern for patients undergoing surgeries as it may require additional hospitalization or clinical management^3–5^. As reported in 2013, the incidence rate of postoperative complication is 37%, and the rate of 30-day postoperative mortality is reported to range from 0.79 to 5.7%, depending on the type of surgery and number of postoperative complications experienced^6–8^. Although the incidence of postoperative mortality has decreased owing to improved preoperative management of surgical patients, postoperative death is the most serious type of postoperative complication and causes a great socioeconomic burden. Accurate prediction of postoperative mortality is important for not only successful postoperative patient care but also for information-based shared decision-making with patients and efficient allocation of medical resources^9,10^.

Although many tools for preoperative risk assessment have been developed, previous risk scoring systems have some limitations. The American Society of Anesthesiologists (ASA) physical status classification (ASA-PS) categorizes patients into six subgroups with different prognosis based on the patients’ physical fitness, where a higher score suggests higher likelihood of experiencing a postoperative complication^11^. However, since the ASA class depends on the subjective intuition of the clinician, there may be inter-observer variability, which is a limitation^11^. The physiologic and operative severity score for the enumeration of mortality and morbidity (POSSUM) has a limitation that manually collected data are needed, and the American College of Surgeons national surgical quality improvement program (ACS-NSQIP) has the disadvantage that it is too complicated to be applied in clinical practice^12–15^. The surgical outcome risk tool (SORT) has the disadvantage of lack of external validation^16^. The Surgical Apgar score is easy to apply but has poor prediction performance^17^. While these risk scoring systems are based on the physician’s history-taking and statistical methods such as logistic or Cox regression, recent breakthroughs in machine learning have led to attempts of using it for predicting postoperative prognosis^18–34^. Furthermore, newer machine-learning models, such as extreme gradient boosting (XGBoost)^35^ and deep neural networks^36^, have demonstrated superior predictive performance on nonlinear data as compared to conventional linear models such as logistic or Cox regression^37^. Because patient wellness is defined in many situations by avoiding extremely low and extremely high levels of medical conditions, the statistical assumption of linear inherence in traditional logistics or Cox regression does not always necessarily be the optimal choice. As a result, the application of innovative machine-learning techniques capable of capturing nonlinearity in clinical practice is imperative.

While machine-learning models have outperformed the previous risk scoring systems, these have limitations in that they are mostly designed on single-centered data and have a fairly large number of input features for clinical application^18–21^. Machine learning has proven its capability in the creation of high-quality prediction models, but it is important to create models that are clinically applicable and robust against heterogeneous populations in the real world.

This study thus aimed to create a machine-learning prediction model for 30-day mortality after a non-cardiac surgery that adapts to a manageable amount of objective and quantitative clinical information as input features and is validated against multi-centered rather than single-centered data. Because this model does not rely on medical staff to subjectively generate data, it minimizes the inaccuracy of input data and does not require more human resources to generate and collect data. Additionally, since the input data is objective, it is possible to construct a model that has optimal transferability among hospitals. We hypothesize that the performance of a lighter model that only uses an appropriately small number of input variables for predicting 30-day mortality after a non-cardiac surgery at multiple institutions will be at least as good as that of the previous complex and heavy artificial-intelligence models that use many clinical input variables. By demonstrating that the model has appropriate predictive power and transferability when applied to multiple hospitals, we attempted to confirm the clinical utility of this model.

## Results

### Study population characteristics

The average age of surgical patients was the lowest in Ewha Woman's University Medical Center (EUMC). Orthopedic surgery was the most common type of surgery in Boramae Hospital (BRMH), whereas general surgery was the most common in the remaining three hospitals. Owing to this difference in the distribution of surgery departments between the hospitals, the proportion of neuro-axial anesthesia, such as spinal anesthesia, in BRMH was higher than those in other hospitals. The rate of emergency surgery was the highest in Asan Medical Center (AMC). The mortality rate within 30 days after surgery was reported to be as low as 0.2–0.4% in all four hospitals. Table 1 presents the data characteristics of each hospital.

**Table 1:** Data characteristics of the four medical institutions.

	SNUH	AMC	EUMC	BRMH	*P* value
	(*n* = 223,905)	(*n* = 66,522)	(*n* = 131,867)	(*n* = 32,110)	
Demographic data					
Age, years	53.7 ± 16.1	54.7 ± 15.9	48.5 ± 17.1	56.7 ± 17.6	<0.001
Sex, female	123,670 (55.2%)	38,117 (57.3%)	79,232 (60.1%)	17,560 (54.7%)	<0.001
Body-mass index, kg/m^2^	23.6 ± 3.6	24.1 ± 3.8	23.8 ± 3.8	24.7 ± 4.0	<0.001
Preoperative laboratory results					
White blood cell, 10^3^/μL	6.61 ± 2.69	6.74 ± 2.58	7.49 ± 3.91	7.09 ± 2.84	<0.001
Hemoglobin, g/dL	13.1 ± 1.8	12.7 ± 1.9	13.1 ± 1.9	13.0 ± 1.9	<0.001
Platelet, 10^3^/μL	241.2 ± 74.3	242.5 ± 74.9	245.6 ± 72.0	248.6 ± 76.5	<0.001
Sodium, mmol/L	140.2 ± 2.7	139.8 ± 2.6	140.7 ± 3.0	138.9 ± 2.8	<0.001
Potassium, mmol/L	4.2 ± 0.4	4.3 ± 0.4	4.2 ± 0.4	4.3 ± 0.4	<0.001
BUN, mg/dL	15.0 ± 7.8	16.1 ± 10.7	13.7 ± 6.9	15.6 ± 9.5	<0.001
Creatinine, mg/dL	0.99 ± 1.07	1.00 ± 1.25	0.92 ± 0.68	0.98 ± 1.18	<0.001
Albumin, g/dL	4.1 ± 0.5	3.7 ± 0.5	4.1 ± 0.6	4.1 ± 0.4	<0.001
GOT, IU/L	24.5 ± 79.0	25.1 ± 29.5	26.5 ± 95.0	28.4 ± 114.4	<0.001
GPT, IU/L	23.4 ± 44.9	22.6 ± 31.3	25.1 ± 50.9	24.5 ± 64.7	<0.001
Glucose, mg/dL	110.6 ± 36.7	114.8 ± 38.7	198.3 ± 243.9	119.8 ± 42.4	<0.001
PT, INR	1.01 ± 0.16	1.01 ± 0.14	1.02 ± 0.38	1.05 ± 0.09	<0.001
aPTT, sec	33.0 ± 6.6	27.4 ± 3.7	26.9 ± 5.4	28.5 ± 5.0	<0.001
Type of surgery, *n*					
General surgery	87,681 (39.2%)	22,115 (33.2%)	40,611 (30.8%)	9470 (29.5%)	<0.001
Otolaryngologic surgery	20,776 (9.3%)	5366 (8.1%)	14,279 (10.8%)	4114 (12.8%)	<0.001
Urologic surgery	29,876 (13.3%)	7517 (11.3%)	9117 (6.9%)	3518 (11.0%)	<0.001
Orthopedic surgery	44,503 (19.9%)	8226 (12.4%)	23,486 (17.8%)	9515 (29.6%)	<0.001
Gynecological surgery	32,396 (14.5%)	11,507 (17.3%)	26,509 (20.1%)	3968 (12.4%)	<0.001
Plastic surgery	8665 (3.9%)	2111 (3.2%)	9788 (7.4%)	1002 (3.1%)	<0.001
Type of anesthesia, n					
General anesthesia	182,263 (81.4%)	53,855 (81.0%)	100,223 (76.0%)	21,797 (67.9%)	<0.001
Neuro-axial anesthesia	37,025 (16.5%)	3597 (5.4%)	10,716 (8.1%)	8591 (26.8%)	<0.001
MAC	3475 (1.6%)	0 (0.0%)	4985 (3.8%)	1717 (5.3%)	<0.001
Regional anesthesia	1134 (0.5%)	295 (0.4%)	509 (0.4%)	5 (0.0%)	<0.001
Emergency surgery, n	8450 (3.8%)	5790 (8.7%)	4208 (3.2%)	1142 (4.5%)	<0.001
30 days mortality, n	612 (0.3%)	159 (0.2%)	316 (0.2%)	113 (0.4%)	0.002

Data were presented as mean ± standard deviation, median (interquartile range), or number (percentage).

*BUN* blood urea nitrogen, *GOT* glutamate oxaloacetate transaminase, *GPT* glutamate pyruvate transaminase, *PT* prothrombin time, *aPTT* activated partial thromboplastin time, *MAC* monitored anesthesia care.

### Missing-value characteristics

The mean proportion of missing values were 3.76, 5.30, 16.33, and 19.66% in Seoul National University Hospital (SNUH), AMC, EUMC, and BRMH, respectively (Supplementary Table 1). The proportion of missing values in EUMC’s body-mass index (BMI), type of anesthesia, ASA-PS grade, and BRMH laboratory tests was relatively high, suggesting that these missing values are related to each hospital’s data storage characteristics. For instance, at EUMC, ASA-PS grade information that was recorded separately from the main database was unable to be linked to the main database due to the de-identified patients’ IDs. The correlation between variables measured as a companion test (e.g., laboratory tests) was high (absolute correlation value ≥0.7), but the correlation was not remarkable in the other cases with absolute correlation values less than 0.7 (Supplementary Fig. 1a). The variables with a high percentage of missing values (The BMI, type of anesthesia, ASA-PS grade in EUMC, and laboratory tests in BRMH) did not demonstrate a strong association with other variables’ missing values (absolute correlation value <0.7). Missing-value events did not show a specific pattern in each hospital, indicating that the missing mechanism for each patient in each hospital was random (Supplementary Fig. 1b). Considering this random pattern of missing-value events and low correlation between missing values of variables, we replaced missing values with median values.

### Results of model performance

**Fig. 1:**
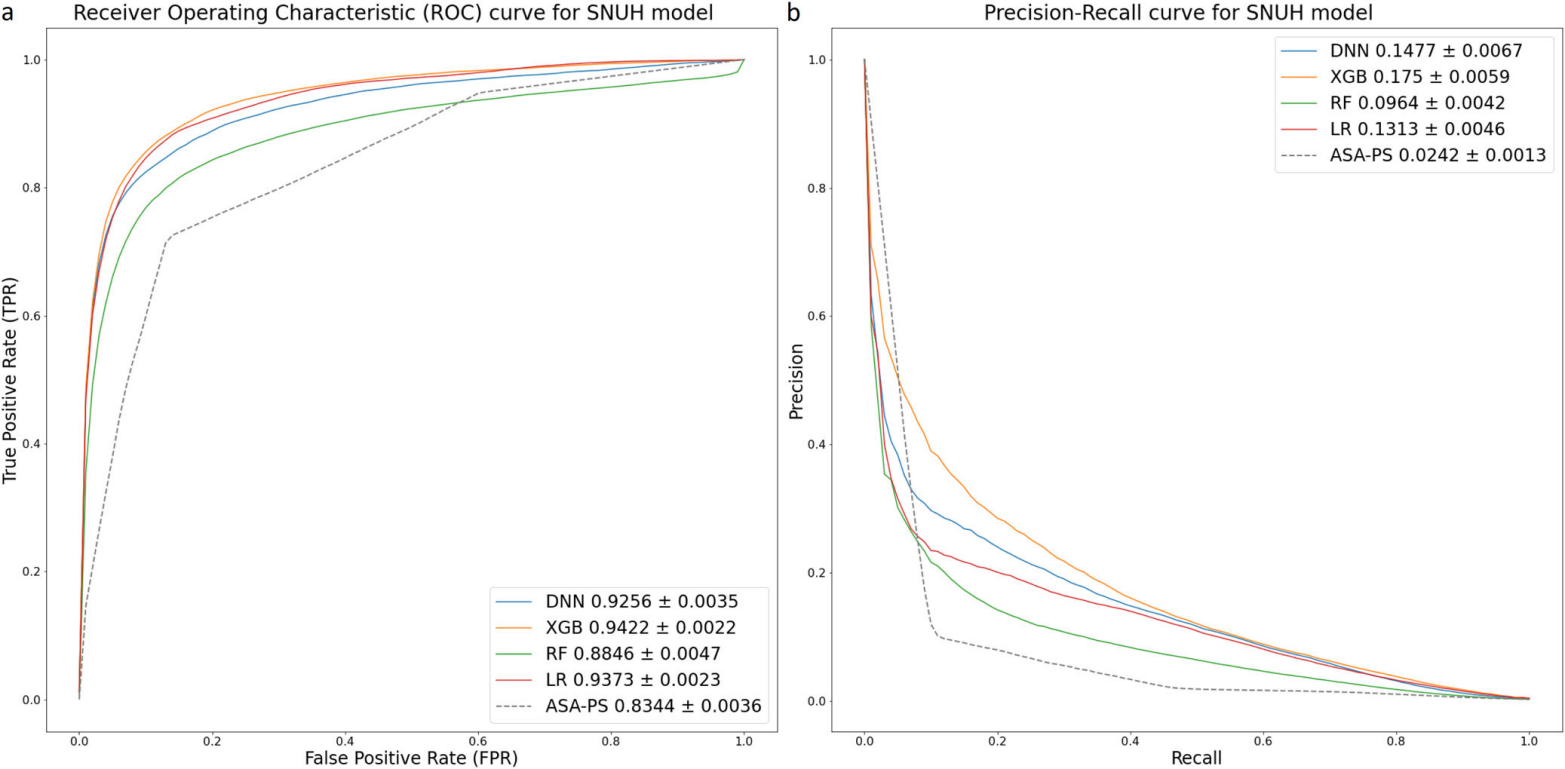
Performance evaluation of machine learning algorithms for postoperative 30-day mortality predictions. AUROC (**a**) and AUPRC (**b**) of several models for postoperative 30-day mortality in the SNUH dataset. The values of AUROC and AUPRC are presented as 95% confidence intervals. AUROC area under receiver operating characteristic curve, AUPRC area under precision-recall curve, DNN deep neural network, XGB extreme gradient boosting, RF random forest, LR logistic regression, ASA-PS American society of anesthesiologists physical status classification.

Figure 1 presents the area under the receiver operating characteristics curve (AUROC) and the area under the precision-recall curve (AUPRC) of each candidate modeling method in SNUH data. All four candidate models exhibited superior prediction performances in terms of AUROC and AUPRC, compared to that of the ASA-PS class. The XGBoost method delivered the best performance in terms of AUROC (0.942) and AUPRC (0.175). The XGBoost method delivered the best performance in the EUMC data, similar to the SNUH model, and the logistic regression (LR) method delivered the best performance in the BRMH data (Supplementary Fig. 2). In the AMC data, the LR model based on AUROC and XGBoost model based on AUPRC delivered the best performance (Supplementary Fig. 2). The XGBoost method delivered the best performance in the lab model of SNUH and EUMC data, as in the conventional model (Supplementary Fig. 3). The LR method delivered the best performance in the AMC lab model. The XGBoost method and LR method delivered the best performances in terms of AUROC and AUPRC, respectively, in the BRMH lab model (Supplementary Fig. 3). Table 2 presents the results of mean values of bootstrapping performance of the conventional models to which the XGBoost algorithm was applied with data from each hospital. Table 2 presents the result obtained using all input variables for the modeling, whereas Table 3 presents the result of comparing them with a lab model using only 12 preoperative variables. Typically, the SNUH model and EUMC model with large amounts of data delivered superior performance when externally validated with data from other institutions. The performance in terms of the AUROC value of external validation is the best when the SNUH model is validated on EUMC data (AUROC 0.941). The performance in terms of the AUPRC value of external validation is the best when the SNUH model is validated on BRMH data (AUPRC 0.180). In the case of external validation of AMC data with the EUMC model in the lab model, the AUROC value was the highest (0.923). When external validation was performed on BRMH data with the SNUH model, the AUPRC value was the highest (0.177).

**Table 2:** Validation results of four models using all features for postoperative 30-day mortality.

Train	Test			
	AUROC			
	SNUH	AMC	EUMC	BRMH
SNUH	0.942 (0.940–0.945)	0.927 (0.923–0.932)	0.941 (0.938–0.943)	0.908 (0.902–0.915)
AMC	0.920 (0.917–0.923)	0.935 (0.930–0.939)	0.915 (0.911–0.919)	0.877 (0.869–0.885)
EUMC	0.934 (0.932–0.937)	0.930 (0.926–0.934)	0.956 (0.953–0.958)	0.912 (0.906–0.919)
BRMH	0.913 (0.910–0.916)	0.895 (0.889–0.901)	0.921 (0.917–0.925)	0.916 (0.909–0.923)
	AUPRC			
SNUH	0.175 (0.169–0.181)	0.096 (0.087–0.104)	0.130 (0.124–0.137)	0.180 (0.166–0.194)
AMC	0.090 (0.086–0.093)	0.092 (0.084–0.100)	0.090 (0.084–0.096)	0.133 (0.121–0.145)
EUMC	0.111 (0.107–0.115)	0.081 (0.074–0.088)	0.155 (0.147–0.163)	0.179 (0.165–0.193)
BRMH	0.105 (0.100–0.109)	0.073 (0.067–0.079)	0.101 (0.094–0.108)	0.142 (0.131–0.153)

Data were presented as means (95% confidence intervals).

*AUROC* area under receiver operating characteristic curve, *AUPRC* area under precision-recall curve, *SNUH* Seoul National University Hospital, *AMC* Asan Medical Center, *EUMC* Ewha Womans University Medical Center, *BRMH* Boramae hospital.

**Table 3:** Validation results of four lab models using only 12 features of laboratory test for postoperative 30-day mortality.

Train	Test			
	AUROC			
	SNUH	AMC	EUMC	BRMH
SNUH	0.926 (0.921–0.930)	0.921 (0.914–0.928)	0.916 (0.910–0.921)	0.888 (0.877–0.899)
AMC	0.912 (0.908–0.916)	0.925 (0.917–0.932)	0.897 (0.891–0.903)	0.861 (0.849–0.873)
EUMC	0.921 (0.917–0.925)	0.923 (0.915–0.930)	0.921 (0.915–0.927)	0.905 (0.895–0.915)
BRMH	0.897 (0.892–0.902)	0.896 (0.887–0.905)	0.891 (0.884–0.898)	0.905 (0.894–0.915)
	AUPRC			
SNUH	0.154 (0.146–0.162)	0.105 (0.092–0.117)	0.125 (0.115–0.135)	0.177 (0.159–0.196)
AMC	0.088 (0.083–0.093)	0.090 (0.079–0.100)	0.086 (0.080–0.093)	0.122 (0.108–0.137)
EUMC	0.115 (0.109–0.122)	0.078 (0.069–0.086)	0.143 (0.133–0.152)	0.163 (0.145–0.182)
BRMH	0.101 (0.095–0.106)	0.079 (0.068–0.090)	0.106 (0.097–0.115)	0.151 (0.134–0.168)

Data were presented as 95% confidence intervals.

*AUROC* area under receiver operating characteristic curve, *AUPRC* area under precision-recall curve, *SNUH* Seoul National University Hospital, *AMC* Asan Medical Center, *EUMC* Ewha Womans University Medical Center, *BRMH* Boramae Hospital.

Supplementary Table 3 presents a comparison of AUROC and AUPRC values between each candidate model in each hospital dataset. In the comparison of AUROC values, the XGBoost model and LR model showed a significant difference between the random forest (RF) model and ASA-PS class in all datasets except for in BRMH dataset. While the XGBoost and LR models of BRMH showed a significant difference to the five-layer deep neural network (DNN) model, this trend was not observed in other datasets. In comparison of AUPRC values, the XGBoost model and LR model showed statistically significant superiority compared to ASA-PS class in all datasets except for in BRMH dataset. The significant differences in AUPRC were observed between the XGBoost model and RF, LR models in the SNUH dataset. The DNN model also presented a significant difference in AUPRC to ASA-PS class in SNUH and EUMC datasets. The DNN model using lab features presented significant difference to RF and LR models (*p* = 0.0196, *p* = 0.0204) in SNUH dataset. Only the LR model using all features showed significant superiority in AUPRC to ASA-PS (*p* = 0.0380) in the BRMH dataset.

Supplementary Fig. 4 presents the results of applying the XGBoost model with tenfold cross-validation trained only the value of the SNUH model to the test data of the four institutions. When the XGBoost model of SNUH was applied to the test data of SNUH using tenfold cross-validation analysis, it was confirmed that the AUPRC value increased from 0.175 to 0.194. When it was applied to AMC, EUMC, and BRMH data, it was confirmed that the AUROC value increased from 0.927 to 0.941, 0.941 to 0.950, and 0.908 to 0.913, respectively. A calibration plot for the tenfold cross-validation model was generated using a test set from SNUH and all datasets of three other hospitals (Supplementary Fig. 5). As a result, in all four hospitals, the mean predictive value and the actual fraction of positives were positively correlated. With increasing mean predictive values in AMC and EUMC, there was a tendency for the mortality rate to be slightly overestimated.

Supplementary Table 2 presents the results of oversampling of positive events and downsampling of negative events at various sampling rates to overcome data imbalance. Compared to reference models that are developed on training data without resampling, models trained with upsampled or downsampled datasets mostly showed a decline in performance. As an exception, the BRMH model reported an increased AUROC for upsampling ratios of 0.01 and 0.1 and downsampling ratios of 0.01, 0.05, 0.1, and 0.25 and an increased AUPRC for upsampling ratios of 0.01 and 0.05 and a downsampling ratio of 0.01. In addition, the EUMC model also reported an increased AUROC of 0.001 in an upsampling ratio of 0.01. No resampling methods increased the AUPRC over the confidence interval of reference.

### Model interpretation via feature importance

**Fig. 2:**
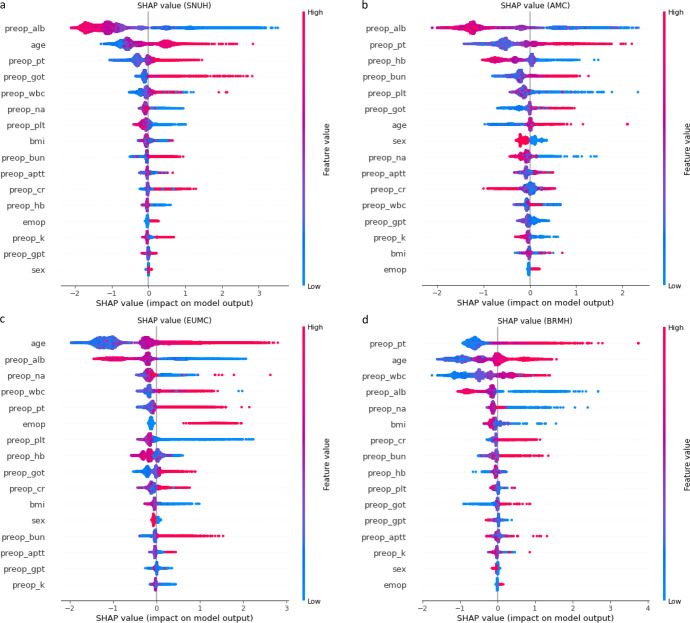
Feature-importance of each hospital model with respect to SHAP value. **a** SNUH model, **b** AMC model, **c** EUMC model, **d** BRMH model. SHAP Shapley additive explanations, SNUH Seoul National University Hospital, AMC Asan Medical Center, EUMC Ewha Womans University Medical Center, BRMH Boramae Hospital.

The feature-importance extracted from the XGBoost algorithm was different for each model (Fig. 2). In the SNUH and AMC models, the preoperative albumin level was found to be variable, with the highest influence on mortality within 30 days after surgery; however, the age in the EUMC model and the preoperative prothrombin time (PT) value in the BRMH model were the most important variables in the postoperative 30-day mortality.

## Discussion

Although numerous artificial intelligence prediction models based on hospital data have been developed to date, their direct application in clinical practice is limited. Existing models required an excessive number of variables in order to enhance prediction performance, and, more crucially, a model developed in one institution functioned poorly in another.

The purpose of this study was to construct a viable artificial intelligence model for predicting prognosis prior to surgery that could be used in the real world. This type of model should exhibit the following characteristics: (1) Models can be transferred between hospitals; (2) data generation and recording do not require additional labor. (3) A straightforward and lightweight design; (4) the accuracy of the model is comparable to that of previous models. To create this model, we used only objective and quantitative data that were automatically imported from the electronic medical record system, reducing interhospital variation and increasing data volume to improve accuracy. The results of this study reveal that prediction power does not decrease even when using only the minimum number of variables that can be automatically extracted from electronic medical records of each hospital, compared to the previously proposed prediction model that requires numerous clinical input variables. Additionally, this model performed well when applied directly to other hospitals, indicating that it is transferrable between hospitals. The disadvantage of existing machine-learning prediction models was that they overfit the training data, making them inapplicable to other hospitals or necessitating retraining. Our model, on the other hand, is directly internalized into the hospital's electronic medical record (EMR) system without additional processing, and prognosis can be predicted with a single click. Even if we do not internalize the program in the EMR, we only use a few parameters, which enables real-time prognosis prediction in an outpatient clinic by entering these parameters into the program via the web.

When the prediction powers of the models developed based on the data of each hospital were compared, it was confirmed that the more the amount of data, the better the prediction power. The prediction model of SNUH, which had the largest amount of data, delivered the best prediction performance. Based on these results, the prediction model developed based on the datasets of merged data of all hospitals would deliver the best prediction performance. However, it is very difficult to implement the merged data by combining datasets from multiple institutions in the real world owing to various legal and institutional regulations. Despite these barriers, this study shows the importance of the amount of data in prediction performance.

From the datasets of the four institutions used in this study, it is shown that the amount and characteristics of each dataset are diversely distributed. Clinically generated data in the real world are hospital-dependent and diversely distributed^38^. Therefore, it is very difficult to develop a robust prediction model applicable to multiple institutions. However, this study showed that a more robust model can be developed with only a small number of variables if a large amount of data are obtained.

In our study, we developed a prediction model using only the results of 12 preoperative laboratory test variables, data of three demographic characteristics, and surgical-related information such as that of the anesthesia method, emergency status, and surgery department. In addition, we developed the lab model using only the results of 12 preoperative laboratory test variables. The reason we performed modeling using only such a small number of variables was to use only objective information that can be commonly extracted from various institutions for applicability to various medical institutions for creating a prediction model. It was expected that the development of a prediction model using only this small number of objective clinical variables would enable the development of a more robust model. As a result, even though only such a small number of variables were used in our models, the prediction performance did not deteriorate, compared to the performances achieved in previous studies. This suggests that a machine-learning model trained only on objective and quantitative values of a sufficiently large cohort may be applied in other hospitals with prediction performance similar to that in the hospital where the model was trained. In a recent study on a deep-learning model that predicts 30-day postoperative mortality, deep-learning techniques such as the convolutional neural network (CNN) and long short-term memory (LSTM) network were developed using a wide range of data including preoperative information as well as intraoperative vital signs and intraoperative drug and fluid data^19^. However, the performance results of the model reveal that AUROC was 0.867 and AUPRC was less than 0.1 in the study^19^.

In our study, the ensemble methods, such as the XGBoost model, delivered the best prediction performance. The XGBoost model delivered superior performance overall, even compared to the performance of the five-layer DNN model. Moreover, in addition to numerical superiority in performance metrics, the XGBoost model presented a statistically significant difference from other prediction models and the ASA-PS model. In comparison to the five-layer DNN model, however, the significance was observed in a part of the datasets. Although the performance results differ depending on the tuning of hyper-parameters, it was confirmed that the XGBoost model delivered performance comparable to that of the deep-learning algorithm in predicting postoperative mortality using preoperative clinical information. Although techniques that can extract feature-importance in deep learning have been recently introduced, the biggest advantage of tree-based machine-learning models is that these can extract the feature-importance of the model, showing the possibility of solving the “black box” problem, which has been reported as the main limitation of artificial-intelligence models^38,39^. In this study, important variables of each hospital model could be determined using Shapley additive explanations (SHAP) values^40^. In SNUH and AMC, albumin was the strongest predictor of postoperative 30-day mortality, whereas, in EUMC and BRMH, age and preoperative PT were the strongest predictors, respectively. Diversity in clinical environment and patient demographics is accountable for the abovementioned variance in feature-importance analysis results of each hospital. Numerous reports have demonstrated that patients with hypoalbuminemia have a poor prognosis following surgery^41^. Serum albumin constitutes 50–70% of total serum protein and is responsible for determining the plasma osmotic pressure. It is produced only in the liver and is significantly decreased in conditions such as liver disease, renal disease, malnutrition, inflammation, and shock^42^. In other words, serum albumin level is a nonspecific factor that represents an overall metabolic function of a patient and hypoalbuminemia prior to surgery indicates a serious illness state, such as considerable inflammation induced by the patient’s disease and impairment of major organ functions, which impairs the body’s ability to recover from surgical stress^43^. Prothrombin is the most abundant coagulation factor in the blood. Prolonged PT may result in substantial bleeding during surgery, which may have a detrimental effect on the prognosis^44^. Since prothrombin is produced by the liver, its abnormality may indicate liver dysfunction^45^. Patients who take warfarin, which prolongs the PT, are more likely to have underlying heart or brain disease. Additionally, if surgery was performed despite prolonged PT, it is highly likely that the patient’s situation was an emergency. Therefore, PT has a strong link with postoperative prognosis. In general, PT may be more indicative of a patient’s overall health than activated partial thromboplastin time (aPTT), as warfarin has no effect on the aPTT and solitary prolonged aPTT is uncommon^46^.

The strength of our study is that it is the only study that collects large-scale data from four independent institutions to create and compare artificial intelligence models that predict postoperative 30-day mortality. In most of the previous studies, models were developed with data from a single-center and validated with data from the same institution. Because of the absence of data for external validation, most of the previous prediction models have overfitting problems, which cause difficulty in applying models developed by one institution to the other. In this situation, our work that externally validates prediction models using multicenter data is expected to serve as an important milestone in the development of generalized, robust models applicable to multiple hospitals.

Because our study excludes intraoperative variables, it has the advantage of being able to predict prognosis in a preoperative setting. If the prognosis is to be predicted in the intensive care unit or postoperative setting, including intraoperative variables, will allow for more accurate prediction. Indeed, when intraoperative data were included, the accuracy of prognosis prediction increased slightly (data not shown). If we incorporate interoperative data into our current model for postoperative care, it is anticipated that we will be able to easily create a model for postoperative care that retains the benefits of our current effective model in the future.

One limitation of our study is the relatively low AUPRC value, compared to the high AUROC value. The AUPRC scores of our models are in the range of 0.1–0.2. As in previous studies, the extremely rare incidence of mortality in each medical center and data imbalance of each hospital may be the cause of the low AUPRC value. Techniques such as oversampling of the incidences and undersampling of the non-incidences were used to attempt to reduce the influence of data imbalance on model performance; however, these techniques failed to improve the AUPRC value. Although the SMOTE that we tried is a popular technique to balance the data, there is a criticism that subsampling has not a theoretical justification and cannot be considered a definitive tool. The researchers pointed out that there is no need to subsample and that only replacing the Bayes rule with the new quantile rule will be theoretically justifiable to solve the class imbalance in data^47^. While a low AUPRC is not uncommon for prediction models in clinical situations, the models we developed achieved better prediction performance than that achieved in previous studies by selecting appropriate modeling methods and training datasets.

The calibration plot indicated a tendency for the final tenfold cross-validation model to slightly overestimate mortality in some hospitals, indicating that clinicians may need to use this model with caution in clinical practice. However, because the death event is the final stage of the clinical aggravation process, a slightly overestimating model would be better than an underestimating model to prevent a patient’s near-death risk situation.

Another limitation of our study is that we did not adopt various technical alternatives for the transferability of the model. Our study confirmed that obtaining as many datasets as possible increases the robustness of the model, but this is very difficult to realize in actual clinical practice. Although it is very important to collect high-quality data and develop a prediction model based on such data to realize a robust model, alternatives for learning imbalanced data with an algorithm such as federated learning have been recently proposed^48,49^. Therefore, various techniques for overcoming this limitation of difficulty in collecting multi-institutional data in the real world are expected to be developed through future research.

It is possible to create a robust artificial intelligence prediction model applicable to multiple institutions through a light predictive model using only minimal preoperative information that can be automatically extracted from each hospital.

## Methods

### Study design

This study was conducted according to “Guidelines for Development and Reporting Machine-Learning Predictive Models in Biomedical Research: A Multidisciplinary View.”^50^ It was approved by the institutional review board (IRB) of Seoul National University Hospital (IRB No. 2011-141-1176), Asan medical center (IRB No. 2021-0186), Ewha Woman's University Hospital (IRB No. 2020-11-017), and Seoul Metropolitan Government-Seoul National University Boramae Medical Center (IRB No. 30-2020-268). Written informed consent was waived owing to the nature of the retrospective study design. This study was executed and reported in accordance with STROBE (STrengthening the Reporting of OBservational studies in Epidemiology) guidelines.

### Inclusion and exclusion criteria

We performed a retrospective analysis of data collected from 454,404 patients over 18 years of age who underwent non-cardiac surgeries from four independent institutions. The dataset of Seoul National University Hospital (SNUH) contains the data of 223,905 patients who underwent surgeries from October 2004 to December 2019, and the dataset of Boramae Hospital (BRMH) contains the data of 32,110 patients who underwent surgeries from January 2016 to December 2019. The dataset of Ewha Womans University Medical Center (EUMC) contains the data of 131,867 patients from January 2001 to December 2019. Data from 66,522 patients who underwent surgeries from March 2019 to April 2021 in Asan Medical Center (AMC) were extracted. We excluded patients who underwent heart surgeries, organ transplant surgeries, and neurosurgeries and excluded patients whose final clinical course was unknown because of a lack of follow-up during the study period.

For patients who underwent multiple surgeries during the study period, only the first surgery performed after admission was included in the analysis.

### Variable selection and data collection

The variable selection process was discussed by all authors, including experts in each clinical department. We listed all available clinical data from the electronic medical record system and performed feature selection (data not shown). The commonly high-ranked features among the four hospitals were considered variable candidates for further analysis. The study omitted variables with major differences between hospitals (e.g., surgery name and diagnosis name) and variables with a high rate of missing values (e.g., troponin I) and a high degree of subjectivity in their measurement and performance by medical staff (e.g., central venous catheterization and arterial catheterization). Finally, we selected the following variables: information on demographics (age, sex, BMI), preoperative laboratory results (white blood cells (WBCs), hemoglobin, platelet, sodium, potassium, blood urea nitrogen (BUN), creatinine, albumin, glutamate oxaloacetate transaminase (GOT), glutamate pyruvate transaminase (GPT), glucose, PT, and aPTT), type of surgery (general, otolaryngologic, urologic, orthopedic, gynecological, and plastic), type of anesthesia (general, neuro-axial, monitored anesthesia care, and regional), emergency surgery were collected.

Data including patient demographics, preoperative laboratory tests, surgical information, and postoperative clinical outcomes were extracted from the electronic medical record system of each hospital. Patient demographic data include age, sex, height, weight, and BMI. Preoperative laboratory tests include WBCs, hemoglobin, platelet, PT, aPTT, sodium, potassium, BUN, creatinine, GOT, GPT, and albumin. Surgical information includes the emergency status of surgery, department of surgery, and type of anesthesia. We also collected the ASA-PS class for each surgical patient evaluated preoperatively.

### Model outcomes

The outcome of the main interest of this study is in-hospital mortality within 30 days after surgery. In-hospital mortality data were extracted as binary information based on the last mortality date in the electronic medical record within 30 days after surgery.

### Model building

Missing values of the model variables were replaced with median values. All continuous variables were scaled using the StandardScaler of Scikit-learn package, and categorical variables were input to the model via one-hot encoding^51^. For modeling, traditional machine-learning methods, such as LR algorithms and RF, were used, and XGBoost ensemble algorithm and DNN methods were applied to compare the model prediction performances^35,36,52,53^. The entire dataset of each hospital was divided into training, validation, and test datasets in a 6:2:2 ratio for the development of all models. The DNN model has a structure consisting of five hidden layers. For each layer of the DNN model, batch normalization and rectified linear unit (ReLu) functions were applied to the dense layer with a dropout rate of 0.5^54^. Cross-entropy was used as the loss function of the model, and the parameters of the models were optimized to minimize the loss function of each model^55^. We used the bootstrap method to overcome the imbalance in clinical data and calculate the performance of each model more robustly. The bootstrap method repeats the process of resampling the training data, training a new model, and evaluating the model multiple times. The performance of the model is then calculated as the average performance of individual models developed by the bootstrap method. Even if a model overfits the data, these problems can be alleviated by averaging their result, yielding a more general model. Bootstrap method can considerably reduce the overfitting of the developed models.

In addition to bootstrap, a tenfold cross-validation method was implemented to generate a final model for clinical application, check the performance of the uniformly trained model on the entire data of one hospital, and confirm the applicability of this model in other hospitals^56^. Grid search was used to find the most optimized hyper-parameter values for the recall, and the training set was trained using a stratified tenfold cross-validation method^57^. The model was tested with the test set of the selected hospital and data from other hospitals to confirm the prediction performance.

We attempted to use two methods, namely upsampling of positive events and downsampling of negative events in the data, to overcome data imbalance for predicting a rare event with an extremely low incidence^58^. First, we upsampled training data for each center using the synthetic minority oversampling technique (SMOTE), so that the ratio of resampled positive events to negative events became 0.01, 0.05, 0.1, or 0.25. We trained XGBoost models with the upsampled data and compared the internal validation results of the models. The second approach was to randomly downsample the training data, so that the ratio of positive events and resampled negative events became 0.01, 0.05, 0.1, or 0.25. Then we compared the internal validation results of the models trained with the downsampled data. We used the SMOTENC function and RandomUnderSampler function of a Python library, imbalanced-learn, to implement SMOTE and random downsampling.

### Model validation

**Fig. 3:**
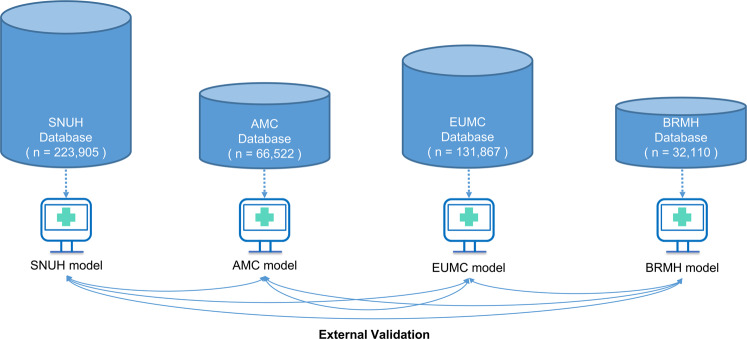
Schematic diagram of external validation of each hospital model. SNUH Seoul National University Hospital, AMC Asan Medical Center, EUMC Ewha Womans University Medical Center, BRMH Boramae Hospital.

Two types of models—a conventional model that uses all input variables and a lab model that only uses results of 12 laboratory test parameters (WBCs, hemoglobin, platelet, PT, aPTT, sodium, potassium, BUN, creatinine, GOT, GPT, and albumin)—were developed, and their performances were compared. The performance of the model was compared with that of the XGBoost model built based on the ASA-PS class. The prediction performance was validated and compared among the local models developed from the data of each hospital. Four local models were developed from the data of four institutions, and each model was externally validated on the data from other hospitals (Fig. 3). The prediction performances were compared in terms of AUROC and AUPRC. The comparison of AUROC and AUPRC was done both numerically and statistically. The statistical comparison of AUROC values and AURPC values was each computed using Delong Test and Permutation test^59–61^ (Supplementary Table 3). A calibration plot was used to evaluate the agreement between the observed and expected values based on the probability of postoperative 30-day mortality predicted by various models.

### Model interpretation via feature-importance

We implemented an explainable AI model by performing a feature-importance analysis on the generated prediction model. The feature-importance of the four independent local models was determined using the Shapley additive explanations (SHAP) values^40^. The SHAP value, which expresses the influence of a variable on a prediction in terms of direction and range, is computed using the prediction outcome of each possible combination of features^40^.

### Statistical analysis and modeling tools

Continuous variables were expressed as means and standard deviations, whereas categorical variables were expressed as numbers and percentages. Continuous variables were compared in four institutions using one-way analysis of variance (ANOVA), whereas categorical variables in the four groups were compared using the Kruskal–Wallis *H*-test. Variables with two-tailed *p* values less than 0.05 were considered statistically significant. Machine learning and DNN modeling was performed in python 3.9 using Scikit-Learn and TensorFlow packages^51^.

### Reporting Summary

Further information on research design is available in the Nature Research Reporting Summary linked to this article.

## Supplementary information

Supplementary information

Reporting Summary

## Data Availability

The dataset used in this study is not publicly available. However, the data of this study can be provided if there is a reasonable request to the corresponding authors.

## Code availability

The code that support the findings of this study are available from the corresponding authors, upon reasonable request.

## References

[1] Weiser TG (2015). Estimate of the global volume of surgery in 2012: an assessment supporting improved health outcomes. Lancet.

[2] Alkire BC (2015). Global access to surgical care: a modelling study. Lancet Glob. Health.

[3] Stephenson C (2020). Management of common postoperative complications. Mayo Clin. Proc..

[4] Anderson O, Davis R, Hanna GB, Vincent CA (2013). Surgical adverse events: a systematic review. Am. J. Surg..

[5] Brennan TA (1991). Incidence of adverse events and negligence in hospitalized patients. Results of the Harvard Medical practice study I. N. Engl. J. Med..

[6] de Vries EN, Ramrattan MA, Smorenburg SM, Gouma DJ, Boermeester MA (2008). The incidence and nature of in-hospital adverse events: a systematic review. Qual. Saf. Health Care.

[7] Tevis SE, Kennedy GD (2013). Postoperative complications and implications on patient-centered outcomes. J. Surg. Res..

[8] Mayo NE (2011). Impact of preoperative change in physical function on postoperative recovery: argument supporting prehabilitation for colorectal surgery. Surgery.

[9] Gunning K, Rowan K (1999). ABC of intensive care: outcome data and scoring systems. BMJ.

[10] Pine M, Norusis M, Jones B, Rosenthal GE (1997). Predictions of hospital mortality rates: a comparison of data sources. Ann. Intern Med..

[11] Cohen ME, Bilimoria KY, Ko CY, Richards K, Hall BL (2009). Effect of subjective preoperative variables on risk-adjusted assessment of hospital morbidity and mortality. Ann. Surg..

[12] Bilimoria KY (2013). Development and evaluation of the universal ACS NSQIP surgical risk calculator: a decision aid and informed consent tool for patients and surgeons. J. Am. Coll. Surg..

[13] Brooks MJ, Sutton R, Sarin S (2005). Comparison of surgical risk score, POSSUM and p-POSSUM in higher-risk surgical patients. Br. J. Surg..

[14] Copeland GP, Jones D, Walters M (1991). POSSUM: a scoring system for surgical audit. Br. J. Surg..

[15] Prytherch DR (1998). POSSUM and Portsmouth POSSUM for predicting mortality. Physiological and operative severity score for the enUmeration of mortality and morbidity. Br. J. Surg..

[16] Protopapa KL, Simpson JC, Smith NC, Moonesinghe SR (2014). Development and validation of the surgical outcome risk tool (SORT). Br. J. Surg..

[17] Gawande AA, Kwaan MR, Regenbogen SE, Lipsitz SA, Zinner MJ (2007). An Apgar score for surgery. J. Am. Coll. Surg..

[18] Chiew CJ, Liu N, Wong TH, Sim YE, Abdullah HR (2020). Utilizing machine learning methods for preoperative prediction of postsurgical mortality and intensive care unit admission. Ann. Surg..

[19] Fritz BA (2019). Deep-learning model for predicting 30-day postoperative mortality. Br. J. Anaesth..

[20] Hill BL (2019). An automated machine learning-based model predicts postoperative mortality using readily-extractable preoperative electronic health record data. Br. J. Anaesth..

[21] Lee CK, Hofer I, Gabel E, Baldi P, Cannesson M (2018). Development and validation of a deep neural network model for prediction of postoperative in-hospital mortality. Anesthesiology.

[22] Seki T, Kawazoe Y, Ohe K (2021). Machine learning-based prediction of in-hospital mortality using admission laboratory data: a retrospective, single-site study using electronic health record data. PLoS One.

[23] Knaus WA, Draper EA, Wagner DP, Zimmerman JE (1985). APACHE II: a severity of disease classification system. Crit. Care Med..

[24] Knaus WA (1991). The APACHE III prognostic system. Risk prediction of hospital mortality for critically ill hospitalized adults. Chest.

[25] Knaus WA, Zimmerman JE, Wagner DP, Draper EA, Lawrence DE (1981). APACHE-acute physiology and chronic health evaluation: a physiologically based classification system. Crit. Care Med..

[26] Zimmerman JE, Kramer AA, McNair DS, Malila FM (2006). Acute physiology and chronic health evaluation (APACHE) IV: hospital mortality assessment for today’s critically ill patients. Crit. Care Med..

[27] Le Gall JR, Lemeshow S, Saulnier F (1993). A new simplified acute physiology score (SAPS II) based on a European/North American multicenter study. JAMA.

[28] Le Gall JR (1984). A simplified acute physiology score for ICU patients. Crit. Care Med..

[29] Moreno RP (2005). SAPS 3-From evaluation of the patient to evaluation of the intensive care unit. Part 2: development of a prognostic model for hospital mortality at ICU admission. Intensive Care Med..

[30] Vincent JL (1996). The SOFA (sepsis-related organ failure assessment) score to describe organ dysfunction/failure. On behalf of the working group on sepsis-related problems of the European society of intensive care medicine. Intensive Care Med..

[31] Deo RC (2015). Machine learning in medicine. Circulation.

[32] Shameer K, Johnson KW, Glicksberg BS, Dudley JT, Sengupta PP (2018). Machine learning in cardiovascular medicine: are we there yet?. Heart.

[33] Redfern OC (2018). Predicting in-hospital mortality and unanticipated admissions to the intensive care unit using routinely collected blood tests and vital signs: development and validation of a multivariable model. Resuscitation.

[34] Rajkomar A (2018). Scalable and accurate deep learning with electronic health records. NPJ Digit. Med..

[35] Chen, T. Q. & Guestrin, C. XGBoost: a scalable tree boosting system. In *Kdd'16: Proceedings of the 22nd Acm Sigkdd International Conference on Knowledge Discovery and Data Mining*. 785–794 (ACM, 2016).

[36] LeCun Y, Bengio Y, Hinton G (2015). Deep learning. Nature.

[37] Goldstein BA, Navar AM, Carter RE (2017). Moving beyond regression techniques in cardiovascular risk prediction: applying machine learning to address analytic challenges. Eur. Heart J..

[38] Johnson, J. M. & Khoshgoftaar,T. M. Survey on deep learning with class imbalance. *J. Big Data* **27** (2019).

[39] Hashimoto DA, Witkowski E, Gao L, Meireles O, Rosman G (2020). Artificial intelligence in anesthesiology: current techniques, clinical applications, and limitations. Anesthesiology.

[40] Lundberg, S. M. & Lee, S.-I. A unified approach to interpreting model predictions. in NIPS'17: Proceedings of the 31st International Conference on Neural Information Processing Systems. (eds von Luxburg, U. et al.) 4765-4774 (Curran Associates Inc., 2017).

[41] Cabrerizo S (2015). Serum albumin and health in older people: review and meta analysis. Maturitas.

[42] Soeters PB, Wolfe RR, Shenkin A (2019). Hypoalbuminemia: pathogenesis and clinical significance. JPEN J. Parenter. Enteral. Nutr..

[43] Scott MJ (2015). Enhanced recovery after surgery (ERAS) for gastrointestinal surgery, part 1: pathophysiological considerations. Acta Anaesth. Scand..

[44] Arnekian V (2012). Use of prothrombin complex concentrate for excessive bleeding after cardiac surgery. Interact. Cardiovasc. Thorac. Surg..

[45] Wada H, Usui M, Sakuragawa N (2008). Hemostatic abnormalities and liver diseases. Semin. Thromb. Hemost..

[46] Lee JWV (2004). Willebrand disease, hemophilia A and B, and other factor deficiencies. Int. Anesthesiol. Clin..

[47] Ishwaran H, O’Brien R (2021). Commentary: the problem of class imbalance in biomedical data. J. Thorac. Cardiovasc. Surg..

[48] Huang L (2019). Patient clustering improves efficiency of federated machine learning to predict mortality and hospital stay time using distributed electronic medical records. J. Biomed. Inf..

[49] Sheller MJ (2020). Federated learning in medicine: facilitating multi-institutional collaborations without sharing patient data. Sci. Rep..

[50] Luo W (2016). Guidelines for developing and reporting machine learning predictive models in biomedical research: a multidisciplinary view. J. Med. Internet Res..

[51] Pedregosa F (2011). Scikit-learn: machine learning in Python. J. Mach. Learn. Res..

[52] Cox DR (1958). The regression analysis of binary sequences. J. R. Stat. Soc. Ser. B (Methodol.).

[53] L B (2001). Random forest. Mach. Learn..

[54] Agarap, A. F. M. Deep learning using rectified linear units (ReLU). Preprint at 10.48550/arXiv.1803.08375 (2019).

[55] Zhang, Z. & Sabuncu, M. R. Generalized cross entropy loss for training deep neural networks with noisy labels. in NIPS'18: Proceedings of the 32nd International Conference on Neural Information Processing Systems. (eds Bengio, S. & Wallach, H. M.) 8792-8802 (Curran Associates Inc., 2018). 10.48550/arXiv.1805.07836. PMC11747755 39839708

[56] Jung Y (2018). Multiple predicting K-fold cross-validation for model selection. J. Nonparametr. Stat..

[57] Shekar, B. H. G. D. *Proc.**2019 Second International Conference on Advanced Computational and Communication Paradigms* (ICACCP). (IEEE, 2019).

[58] Chawla NV, Bowyer KW, Hall LO, Kegelmeyer WP (2002). SMOTE: synthetic minority over-sampling technique. J. Artif. Intell. Res..

[59] Delong ER, Delong DM, Clarkepearson DI (1988). Comparing the areas under 2 or more correlated receiver operating characteristic curves - a nonparametric approach. Biometrics.

[60] Sun X, Xu WC (2014). Fast implementation of DeLong’s algorithm for comparing the areas under correlated receiver operating characteristic curves. IEEE Signal Proc. Let..

[61] D. S. Moore, G. P. McCabe, W. M. Duckworth, S. L. Sclove. *The Practice of Business Statistics: Companion Chapter 18, Bootstrap Methods and Permutation Tests* 1st edn (W. H. Freeman, 2002).

