# Supplementary material for: Multi-center validation of machine learning model for preoperative prediction of postoperative mortality
Source: NPJ Digit Med. 2022 Jul 12;5:91. doi: 10.1038/s41746-022-00625-6 (PMC9276734; doi:10.1038/s41746-022-00625-6)
Supplement: Supplementary file 1 — Supplementary information [file 41746_2022_625_MOESM1_ESM.pdf]

Supplementary information

Multi-center validation of machine learning model for preoperative prediction of postoperative mortality

Seung Wook Lee BS[1], Hyung-Chul Lee MD PhD[2], Jungyo Suh MD[3], Kyung Hyun Lee BS[4], Heonyi Lee MS[5], Suryang Seo ME[6], Tae Kyong Kim MD PhD[7], Sang-Wook Lee MD PhD[8]*, Yi-Jun Kim MD PhD[9]*.

1. School of Medicine, Kyungpook National University, Daegu, Republic of Korea
2. Department of Anesthesiology and Pain Medicine, Seoul National University Hospital, Seoul National University College of Medicine, Seoul, Republic of Korea
3. Department of Urology, Asan Medical Center, University of Ulsan College of Medicine, Seoul, Republic of Korea
4. Department of Digital Health, SAIHST, Sungkyunkwan University, Seoul, Republic of Korea
5. Bioinformatics Collaboration Unit, Department of Biomedical Systems informatics, Yonsei University College of medicine, Seoul, Republic of Korea
6. Department of Nursing, SMG-SNU Boramae Medical Center, Seoul National University College of Medicine, Seoul, South Korea
7. Department of Anesthesiology and Pain Medicine, SMG-SNU Boramae Medical Center, Seoul National University College of Medicine, Seoul, South Korea
8. Department of Anesthesiology and Pain Medicine, Asan Medical Center, University of Ulsan College of Medicine, Seoul, Republic of Korea
9. Institute of Convergence Medicine, Ewha Womans University Mokdong Hospital, Seoul, Republic of Korea

Contents

Supplementary Table 1. Missing value characteristics

Supplementary Table 2.(a) Internal validation result of models trained with incidence-upsampled training data using all features for postoperative 30-day mortality

Supplementary Table 2.(b) Internal validation result of models trained with non-incidence downsampled training data using all features for postoperative 30-day mortality

Supplementary Table 3.(a) Comparison of AUROC values between several models for postoperative 30-day mortality in each hospital dataset

Supplementary Table 3.(b) Comparison of AUPRC values between several models for postoperative 30-day mortality in each hospital dataset

Supplementary Figure 1.(a) Nullity Correlation Heatmap of in each hospital

Supplementary Figure 1.(b) Distribution of missing values and data completeness of dataset in each hospital

Supplementary Figure 2.(a) AUROC and AUPRC of several models for postoperative 30-day mortality in AMC dataset

Supplementary Figure 2.(b) AUROC and AUPRC of several models for postoperative 30-day mortality in EUMC dataset

Supplementary Figure 2.(c) AUROC and AUPRC of several models for postoperative 30-day mortality in BRMH dataset

Supplementary Figure 3.(a) AUROC and AUPRC of lab models for postoperative 30-day mortality in SNUH dataset

Supplementary Figure 3.(b) AUROC and AUPRC of lab models for postoperative 30-day mortality in AMC dataset

Supplementary Figure 3.(c) AUROC and AUPRC of lab models for postoperative 30-day mortality in EUMC dataset

Supplementary Figure 3.(d) AUROC and AUPRC of lab models for postoperative 30-day mortality in BRBH dataset

Supplementary Figure 4. ROC and PR curves of XGBoost model of SNUH with 10-fold cross-validation and its test results on the SNUH test set, AMC, EUMC, and BRMH dataset, respectively.

Supplementary Figure 5. Calibration curve of XGBoost model of SNUH with 10-fold cross validation on the SNUH test set, AMC, EUMC, and BRMH dataset.

Supplementary Table 1. Missing value characteristics

Characteristics	SNUH (n=223,905)			AMC (n=66,522)			EUMC (n=131,867)			BRMH (n=32,110)		
	Available Data	Missing data	Missing Data (%)	Available Data	Missing data	Missing Data (%)	Available Data	Missing data	Missing Data (%)	Available Data	Missing data	Missing Data (%)
Demographics												
Age	223,905	0	0.00	66,519	3	0.00	131,867	0	0.00	32,110	0	0.00
Sex	223,905	0	0.00	66,519	3	0.00	131,867	0	0.00	32,110	0	0.00
Body Mass Index (BMI)	219,526	4,379	1.96	60,912	5,610	8.43	53,819	78,048	59.19	31,821	289	0.90
Preoperative Laboratory Tests												
White Blood Cells	214,808	9,097	4.06	64,750	1,772	2.66	131,761	106	0.08	30,287	1,823	5.68
Hemoglobin	214,824	9,081	4.06	64,750	1,772	2.66	131,867	0	0.00	21,496	10,614	33.06
Platelet	214,568	9,337	4.17	64,750	1,772	2.66	131,757	110	0.08	21,491	10,619	33.07
Prothrombin Time (PT)	209,293	14,612	6.53	60,766	5,756	8.65	116,473	15,394	11.67	29,758	2,352	7.32
Activated Partial Thromboplastin Time (aPTT)	208,823	15,082	6.74	60,375	6,147	9.24	116,945	14,922	11.32	21,382	10,728	33.41
Sodium	214,551	9,354	4.18	63,372	3,150	4.74	123,399	8,468	6.42	21,207	10,903	33.96
Potassium	214,591	9,314	4.16	63,372	3,150	4.74	123,400	8,467	6.42	21,206	10,904	33.96
Blood Urea Nitrogen (BUN)	211,146	12,759	5.70	61,420	5,102	7.67	127,193	4,674	3.54	21,386	10,724	33.40
Creatinine	211,213	12,692	5.67	64,381	2,141	3.22	127,394	4,473	3.39	21,401	10,709	33.35
Glutamate Oxaloacetate Transaminase (GOT)	210,886	13,019	5.81	64,766	1,756	2.64	127,541	4,326	3.28	21,397	10,713	33.36
Glutamate Pyruvate Transaminase (GPT)	210,892	13,013	5.81	64,766	1,756	2.64	127,539	4,328	3.28	21,397	10,713	33.36
Albumin	210,635	13,270	5.93	64,724	1,798	2.70	108,198	23,669	17.95	21,303	10,807	33.66
Surgical Informations												
Emergency status of surgery	223,503	402	0.18	66,519	3	0.00	131,867	0	0.00	25,347	6,763	21.06
Department of Surgery	223,897	8	0.00	57,623	8,899	13.38	131,867	0	0.00	32,099	11	0.03
Type of Anesthesia	223,897	8	0.00	57,747	8,775	13.19	21,525	110,342	83.68	32,110	0	0.00
ASA-PS Class	209,215	14,690	6.56	58,881	7,641	11.49	0	131,867	100.00	30,831	1,279	3.98
Mean of missing data (%)			3.76			5.30			16.33			19.66

SNUH, Seoul national university hospital; AMC, Asan medical center; EUMC, Ewha womans university medical center; BRMH, Boramae hospital.

Supplementary Table 2.(a) Internal validation result of models trained with incidence-upsampled training data using all features for postoperative 30-day mortality

		Upsampling ratio (N_{rm}/N_M)			
Center	Reference	AUROC			
		0.01	0.05	0.1	0.25
SNUH	0.942 (0.940-0.945)	0.939 (0.934-0.945)	0.942 (0.938-0.946)	0.936 (0.931-0.94)	0.936 (0.933-0.94)
AMC	0.935 (0.930-0.939)	0.932 (0.924-0.941)	0.934 (0.924-0.943)	0.94 (0.932-0.947)	0.925 (0.918-0.931)
EUMC	0.956 (0.953-0.958)	0.957 (0.953-0.961)	0.954 (0.949-0.959)	0.956 (0.952-0.961)	0.949 (0.945-0.952)
BRMH	0.916 (0.909-0.923)	0.917 (0.906-0.929)	0.911 (0.897-0.925)	0.928 (0.918-0.938)	0.903 (0.894-0.912)
Center	Reference	AUPRC			
		0.01	0.05	0.1	0.25
SNUH	0.175 (0.169-0.181)	0.158 (0.145-0.171)	0.156 (0.145-0.168)	0.139 (0.129-0.149)	0.13 (0.122-0.137)
AMC	0.092 (0.084-0.100)	0.088 (0.075-0.1)	0.091 (0.075-0.107)	0.079 (0.069-0.09)	0.071 (0.061-0.081)
EUMC	0.155 (0.147-0.163)	0.145 (0.131-0.16)	0.139 (0.125-0.153)	0.133 (0.117-0.149)	0.11 (0.101-0.118)
BRMH	0.142 (0.131-0.153)	0.147 (0.121-0.172)	0.143 (0.121-0.165)	0.133 (0.11-0.156)	0.128 (0.11-0.146)

Supplementary Table 2.(b) Internal validation result of models trained with non-incidence downsampled training data using all features for postoperative 30-day mortality

		Downsampling ratio (N_m/N_{rM})			
Center	Reference	AUROC			
		0.01	0.05	0.1	0.25
SNUH	0.942 (0.940-0.945)	0.94 (0.935-0.946)	0.941 (0.936-0.945)	0.944 (0.94-0.949)	0.94 (0.935-0.945)
AMC	0.935 (0.930-0.939)	0.935 (0.924-0.945)	0.93 (0.918-0.941)	0.933 (0.924-0.941)	0.927 (0.919-0.935)
EUMC	0.956 (0.953-0.958)	0.955 (0.951-0.959)	0.954 (0.949-0.959)	0.953 (0.949-0.957)	0.954 (0.95-0.959)
BRMH	0.916 (0.909-0.923)	0.921 (0.905-0.937)	0.929 (0.917-0.94)	0.927 (0.917-0.937)	0.923 (0.912-0.934)
Center	Reference	AUPRC			
		0.01	0.05	0.1	0.25
SNUH	0.175 (0.169-0.181)	0.174 (0.164-0.185)	0.15 (0.141-0.159)	0.149 (0.138-0.159)	0.126 (0.116-0.137)
AMC	0.092 (0.084-0.100)	0.089 (0.077-0.102)	0.089 (0.073-0.104)	0.087 (0.073-0.1)	0.069 (0.058-0.08)
EUMC	0.155 (0.147-0.163)	0.145 (0.131-0.16)	0.139 (0.123-0.154)	0.115 (0.105-0.126)	0.107 (0.094-0.119)
BRMH	0.142 (0.131-0.153)	0.147 (0.12-0.174)	0.129 (0.105-0.153)	0.123 (0.099-0.146)	0.103 (0.086-0.119)

Data are presented as means (95% confidence intervals).

AUROC, area under receiver operating characteristic curve; AUPRC, area under precision-recall curve; N_{rm}, number of samples reporting postoperative 30-day mortality after resampling; N_M, number of samples not reporting postoperative 30-day mortality; N_m, number of samples reporting postoperative 30-day mortality; N_{rM}, number of samples not reporting postoperative 30-day mortality after resampling; SNUH, Seoul national university hospital; AMC, Asan medical center; EUMC, Ewha womans university medical center; BRMH, Boramae hospital.

Supplementary Table 3. (a) Comparison of AUROC values between several models for postoperative 30-day mortality in each hospital dataset

SNUH model using all features

	XGB	DNN	RF	LR	ASA-PS
**XGB**		0.5163	0.0480	0.4636	0.0000
**DNN**	0.5163		0.0881	0.3851	0.0000
**RF**	0.0480	0.0881		0.0254	0.0816
**LR**	0.4636	0.3851	0.0254		0.0000
**ASA-PS**	0.0000	0.0000	0.0816	0.0000	

SNUH models using lab features

	XGB	DNN	RF	LR	ASA-PS
**XGB**		0.3187	0.0262	0.2039	0.0000
**DNN**	0.3187		0.1746	0.5833	0.0013
**RF**	0.0262	0.1746		0.1221	0.0609
**LR**	0.2039	0.5833	0.1221		0.0007
**ASA-PS**	0.0000	0.0013	0.0609	0.0007	

AMC model using all features

	XGB	DNN	RF	LR	ASA-PS
**XGB**		0.0547	0.0070	0.5987	0.0016
**DNN**	0.0547		0.1839	0.0949	0.3697
**RF**	0.0070	0.1839		0.0071	0.4331
**LR**	0.5987	0.0949	0.0071		0.0066
**ASA-PS**	0.0016	0.3697	0.4331	0.0066	

AMC models using lab features

	XGB	DNN	RF	LR	ASA-PS
**XGB**		0.3140	0.0291	0.4782	0.0017
**DNN**	0.3140		0.2120	0.3517	0.1128
**RF**	0.0291	0.2120		0.0832	0.3534
**LR**	0.4782	0.3517	0.0832		0.0106
**ASA-PS**	0.0017	0.1128	0.3534	0.0106	

EUMC models using all features

	XGB	DNN	RF	LR
**XGB**		0.1730	0.0129	0.5933
**DNN**	0.1730		0.2981	0.1763
**RF**	0.0129	0.2981		0.0312
**LR**	0.5933	0.1763	0.0312	

EUMC models using lab features

	XGB	DNN	RF	LR
**XGB**		0.4131	0.0367	0.5083
**DNN**	0.4131		0.0695	0.2799
**RF**	0.0367	0.0695		0.0986
**LR**	0.5083	0.2799	0.0986	

BRMH models using all features

	XGB	DNN	RF	LR	ASA-PS
**XGB**		0.0395	0.0394	0.4909	0.1219
**DNN**	0.0395		0.4882	0.0190	0.3175
**RF**	0.0394	0.4882		0.0242	0.2661
**LR**	0.4909	0.0190	0.0242		0.0804
**ASA-PS**	0.1219	0.3175	0.2661	0.0804	

BRMH models using lab features

	XGB	DNN	RF	LR	ASA-PS
**XGB**		0.0416	0.0991	0.6180	0.3140
**DNN**	0.0416		0.5181	0.0332	0.2563
**RF**	0.0991	0.5181		0.1094	0.3268
**LR**	0.6180	0.0332	0.1094		0.3642
**ASA-PS**	0.3140	0.2563	0.3268	0.3642	

Supplementary Table 3. (b) Comparison of AUPRC values between several models for postoperative 30-day mortality in each hospital dataset

SNUH model using all features					
	**XGB**	**DNN**	**RF**	**LR**	**ASA-PS**
**XGB**		0.3032	0.0240	0.0312	0.0004
**DNN**	0.7012		0.0808	0.1236	0.0004
**RF**	0.9768	0.9256		0.7824	0.0016
**LR**	0.9736	0.8748	0.1944		0.0000
**ASA-PS**	1.0000	1.0000	0.9988	1.0000	

SNUH models using lab features					
	**XGB**	**DNN**	**RF**	**LR**	**ASA-PS**
**XGB**		0.3040	0.0060	0.0144	0.0000
**DNN**	0.6896		0.0196	0.0204	0.0004
**RF**	0.9900	0.9752		0.7204	0.0024
**LR**	0.9812	0.9712	0.2800		0.0004
**ASA-PS**	1.0000	0.9996	0.9984	1.0000	

AMC model using all features					
	**XGB**	**DNN**	**RF**	**LR**	**ASA-PS**
**XGB**		0.1036	0.0744	0.3360	0.0220
**DNN**	0.9036		0.3712	0.8356	0.1028
**RF**	0.9364	0.6200		0.8756	0.1816
**LR**	0.6428	0.1540	0.1268		0.0356
**ASA-PS**	0.9692	0.8968	0.8040	0.9584	

AMC models using lab features					
	**XGB**	**DNN**	**RF**	**LR**	**ASA-PS**
**XGB**		0.2660	0.1680	0.6064	0.0436
**DNN**	0.7300		0.3364	0.7860	0.0620
**RF**	0.8288	0.6292		0.8924	0.1348
**LR**	0.3856	0.1984	0.1236		0.0428
**ASA-PS**	0.9608	0.9444	0.8624	0.9608	

EUMC models using all features*				
	**XGB**	**DNN**	**RF**	**LR**
**XGB**		0.2676	0.1460	0.3676
**DNN**	0.7416		0.2988	0.6128
**RF**	0.8512	0.6848		0.8024
**LR**	0.6244	0.4012	0.2024	

EUMC models using lab features*				
	**XGB**	**DNN**	**RF**	**LR**
**XGB**		0.6272	0.1328	0.2972
**DNN**	0.3812		0.0924	0.2060
**RF**	0.8504	0.9112		0.6828
**LR**	0.7048	0.7920	0.3272	

BRMH models using all features					
	**XGB**	**DNN**	**RF**	**LR**	**ASA-PS**
**XGB**		0.4868	0.5148	0.7988	0.0796
**DNN**	0.5116		0.5356	0.8224	0.1384
**RF**	0.4852	0.4600		0.7600	0.1156
**LR**	0.1980	0.1708	0.2420		0.0380
**ASA-PS**	0.9084	0.8572	0.8696	0.9532	

BRMH models using lab features					
	**XGB**	**DNN**	**RF**	**LR**	**ASA-PS**
**XGB**		0.2868	0.2016	0.5808	0.0796
**DNN**	0.7160		0.4576	0.8332	0.1848
**RF**	0.7960	0.5656		0.8372	0.1744
**LR**	0.4160	0.1796	0.1636		0.0556
**ASA-PS**	0.9208	0.8256	0.8180	0.9300	

AUROC, area under receiver operating characteristic curve; AUPRC, area under precision-recall curve; DNN, deep neural network; XGB, extreme gradient boosting; RF, random forest; LR, logistic regression; ASA-PS, American society of anesthesiologists physical status classification; SNUH, Seoul national university hospital; AMC, Asan medical center; EUMC, Ewha womans university medical center; BRMH, Boramae hospital

Lab features: Preoperative laboratory data; Consisted of white blood cells (WBCs), hemoglobin, platelet, prothrombin time (PT), activated partial thromboplastin time (aPTT), sodium, potassium, blood urea nitrogen (BUN), creatinine, glutamate oxaloacetate transaminase (GOT), glutamate pyruvate transaminase (GPT), and albumin.

All features: Preoperative laboratory data, patient demographics, and surgical information; Consisted of every lab feature, age, sex, height, weight, BMI, status of surgery, department of surgery, and type of anesthesia (general anesthesia, regional anesthesia).

*ASA-PS model is not provided in EUMC dataset due to absence of ASA-PS in the dataset.

Supplementary Figure 1.(a) Nullity Correlation Heatmap of in each hospital

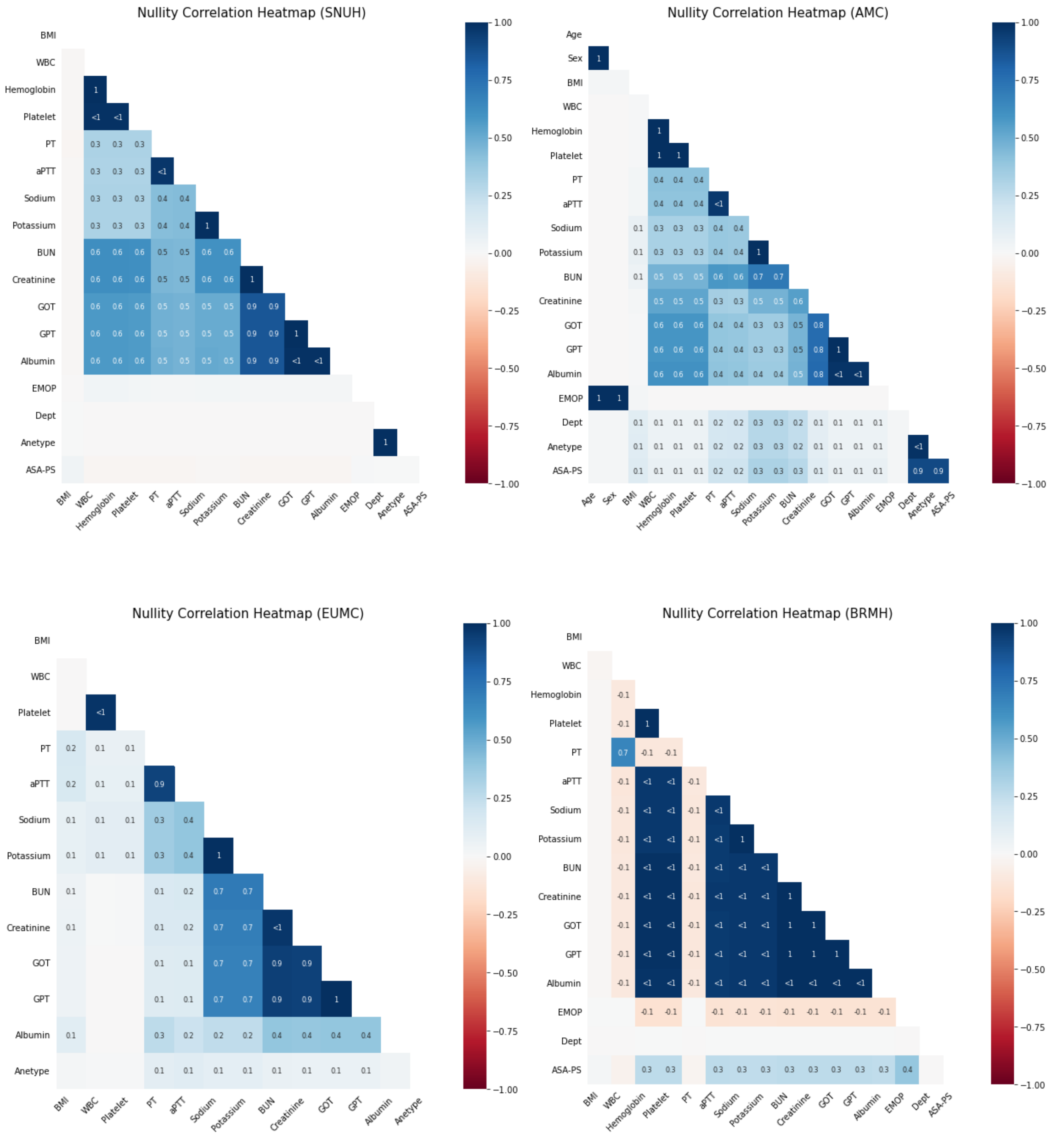

Supplementary Figure 1.(b) Distribution of missing values and data completeness of dataset in each hospital

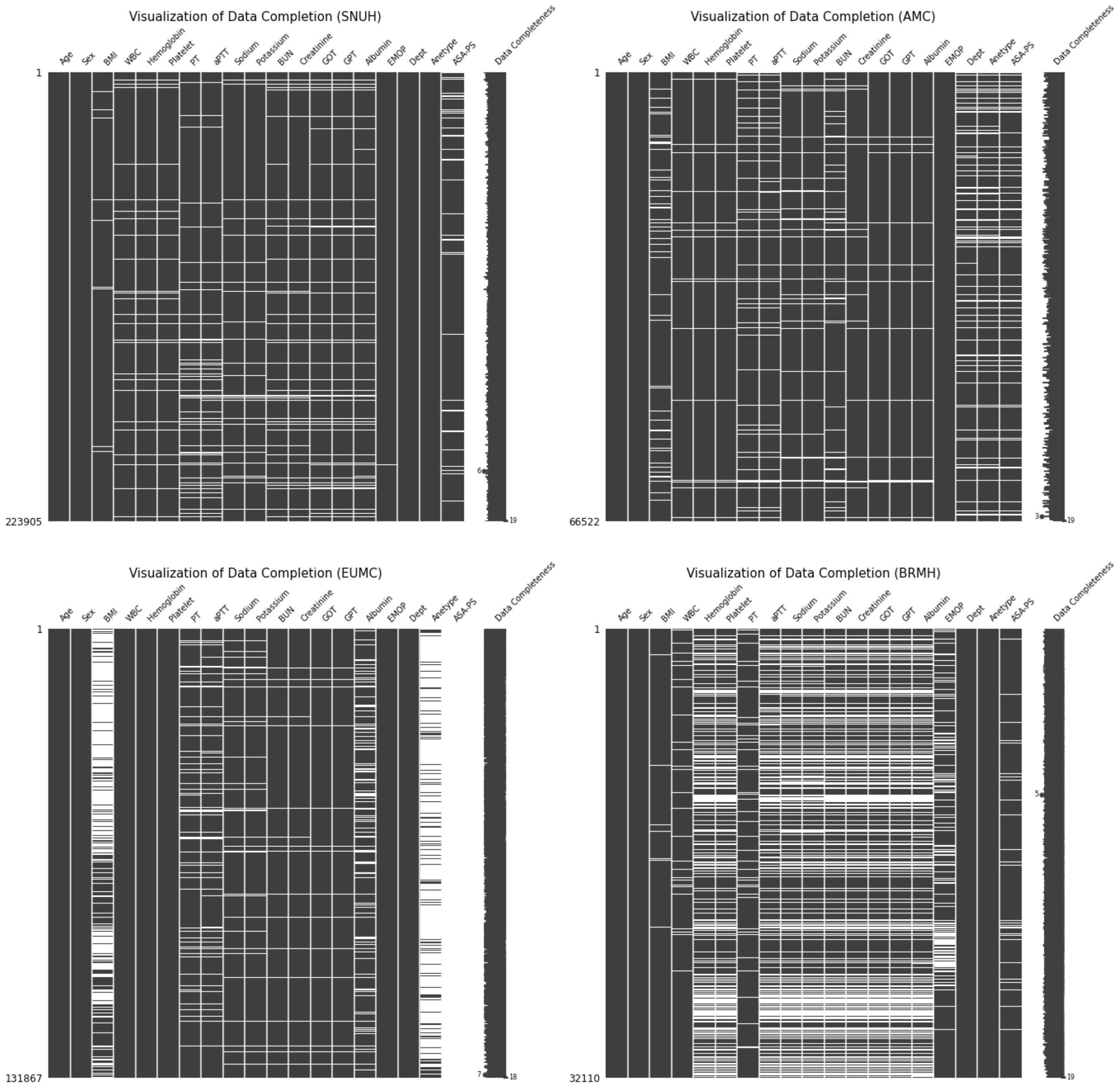

Supplementary Figure 2.(a) AUROC and AUPRC of several models for postoperative 30-day mortality in AMC dataset

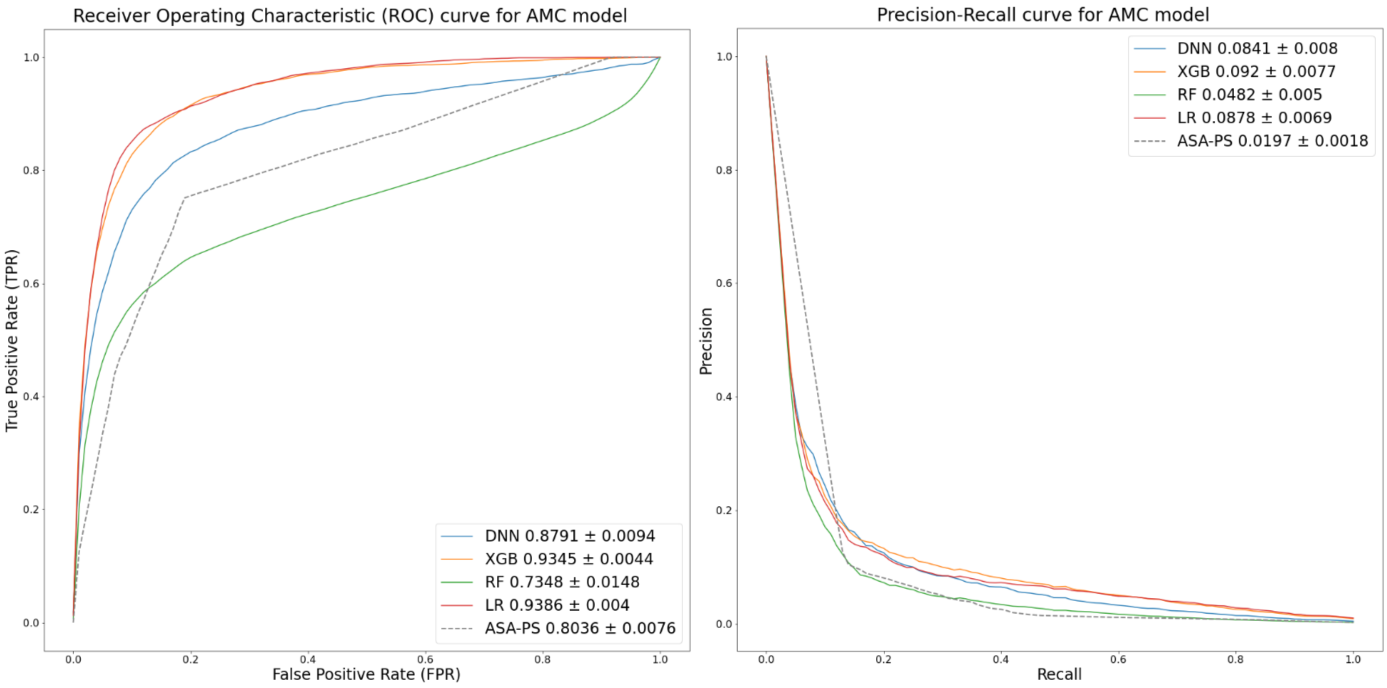

Supplementary Figure 2.(b) AUROC and AUPRC of several models for postoperative 30-day mortality in EUMC dataset. The ASA-PS line is omitted due to the absence of ASA-PS data in EUMC.

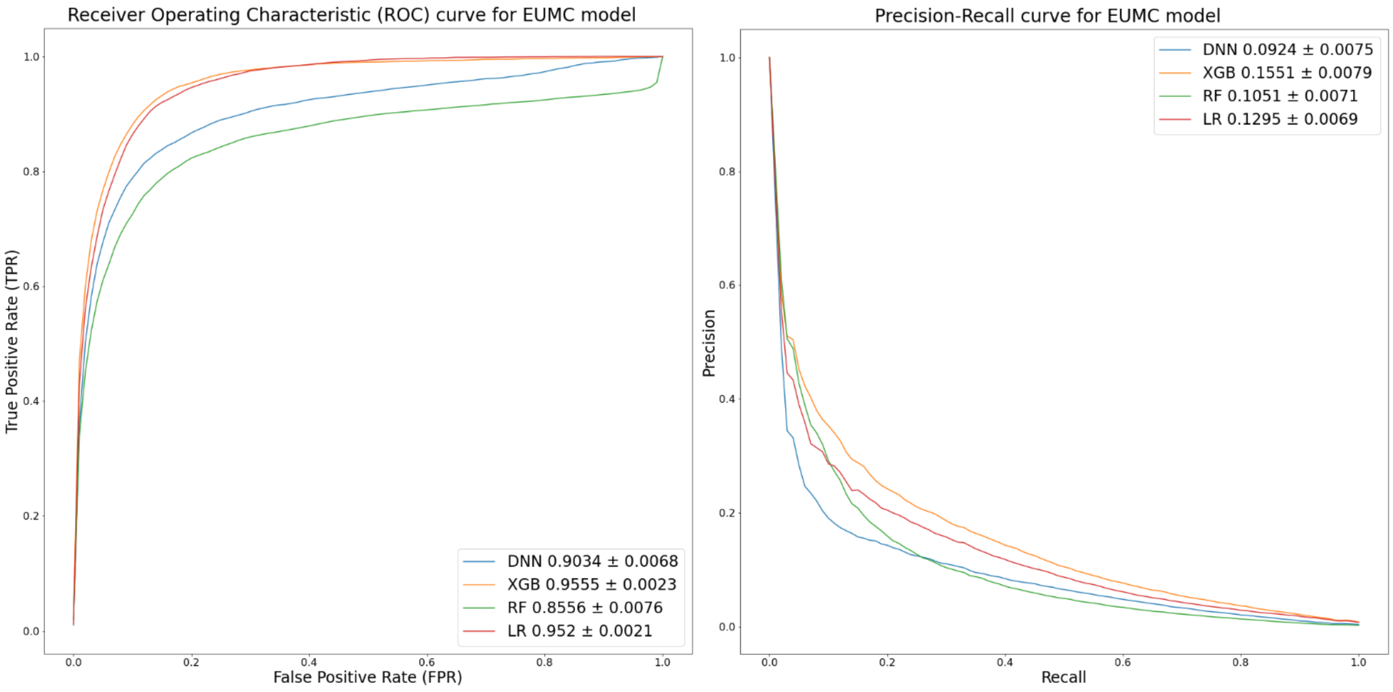

Supplementary Figure 2.(c) AUROC and AUPRC of several models for postoperative 30-day mortality in BRMH dataset

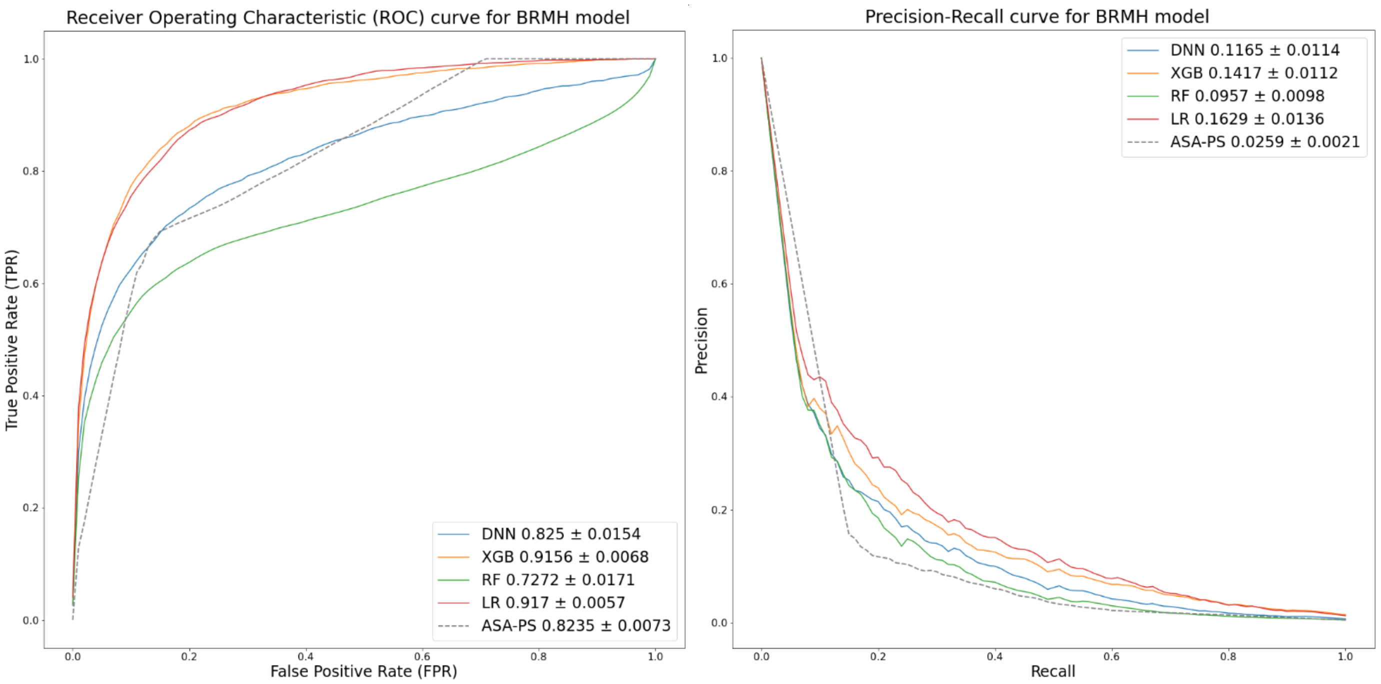

AUROC, area under receiver operating characteristic curve; AUPRC, area under precision-recall curve; DNN, deep neural network; XGB, extreme gradient boosting; RF, random forest; LR, logistic regression; ASA-PS, americanAmerican society of anesthesiologists physical status classification; AMC, Asan medical center; EUMC, Ewha womans university medical center; BRMH, Boramae hospital.

Supplementary Figure 3.(a) AUROC and AUPRC of lab models for postoperative 30-day mortality in SNUH dataset

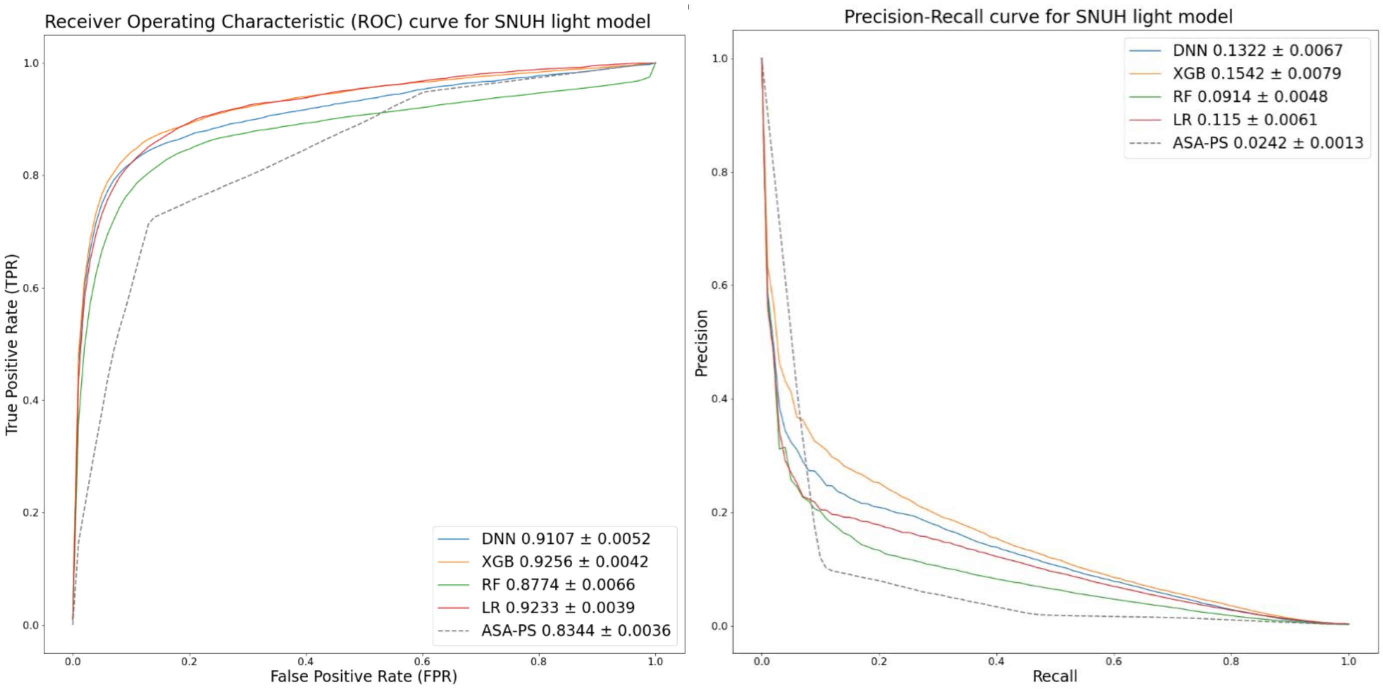

Supplementary Figure 3.(b) AUROC and AUPRC of lab models for postoperative 30-day mortality in AMC dataset

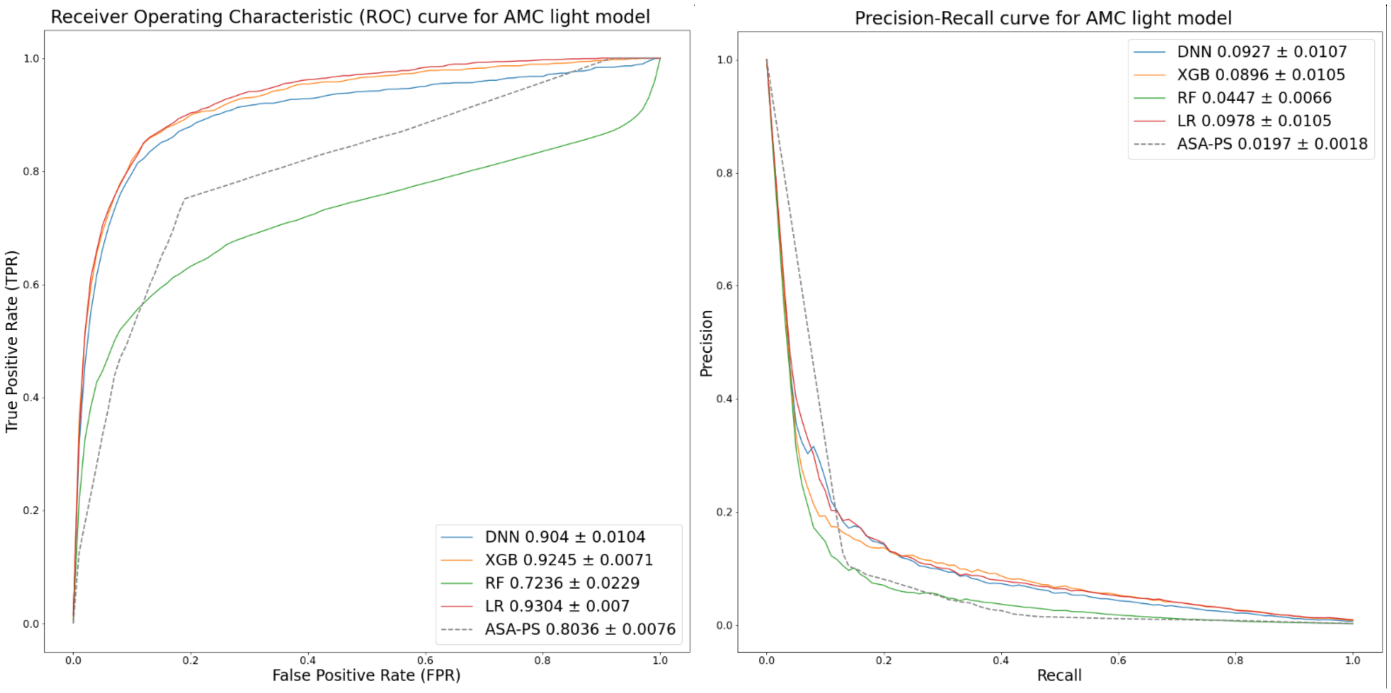

Supplementary Figure 3.(c) AUROC and AUPRC of lab models for postoperative 30-day mortality in EUMC dataset. The ASA-PS line is omitted due to the absence of ASA-PS data in EUMC.

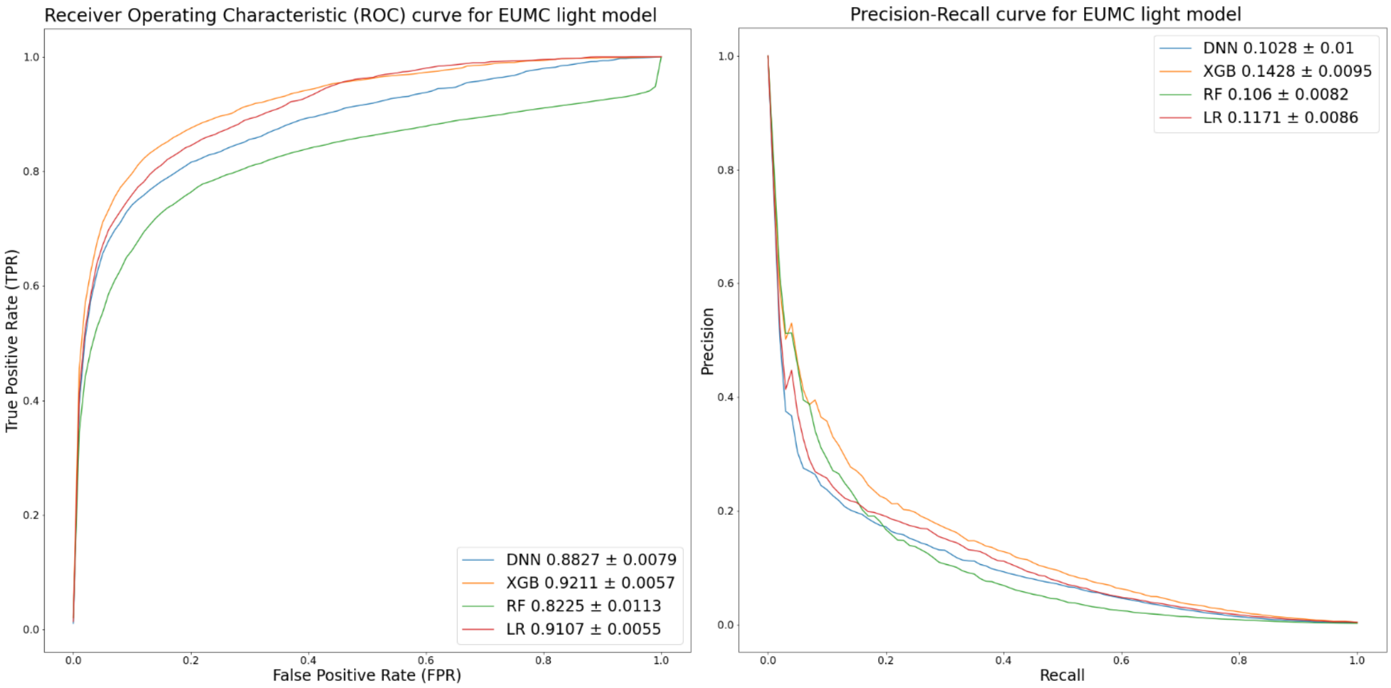

Supplementary Figure 3.(d) AUROC and AUPRC of lab models for postoperative 30-day mortality in BRBH dataset

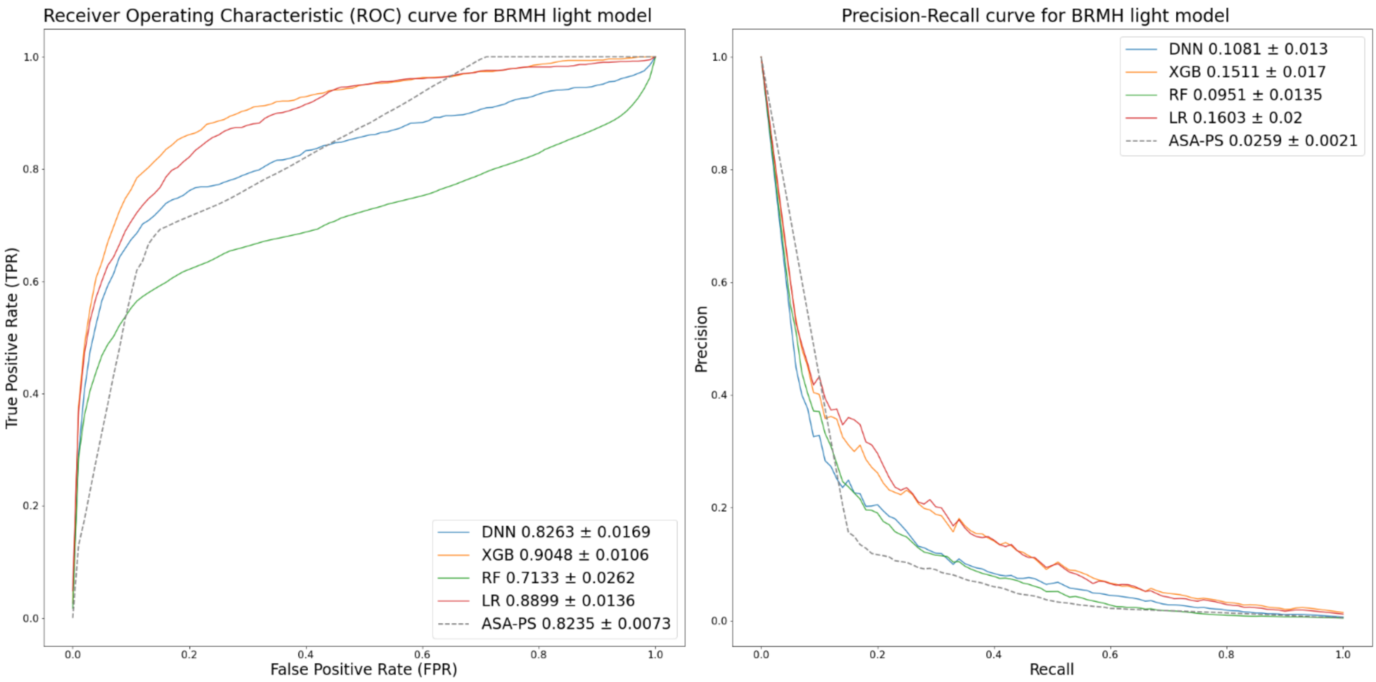

AUROC, area under receiver operating characteristic curve; AUPRC, area under precision-recall curve; DNN, deep neural network; XGB, extreme gradient boosting; RF, random forest; LR, logistic regression; ASA-PS, americanAmerican society of anesthesiologists physical status classification; SNUH, Seoul national university hospital; AMC, Asan medical center; EUMC, Ewha womans university medical center; BRMH, Boramae hospital.

Supplementary Figure 4. ROC and PR curves of XGBoost model of SNUH with 10-fold cross validation and its test results on the SNUH test set, AMC, EUMC, and BRMH dataset, respectively.

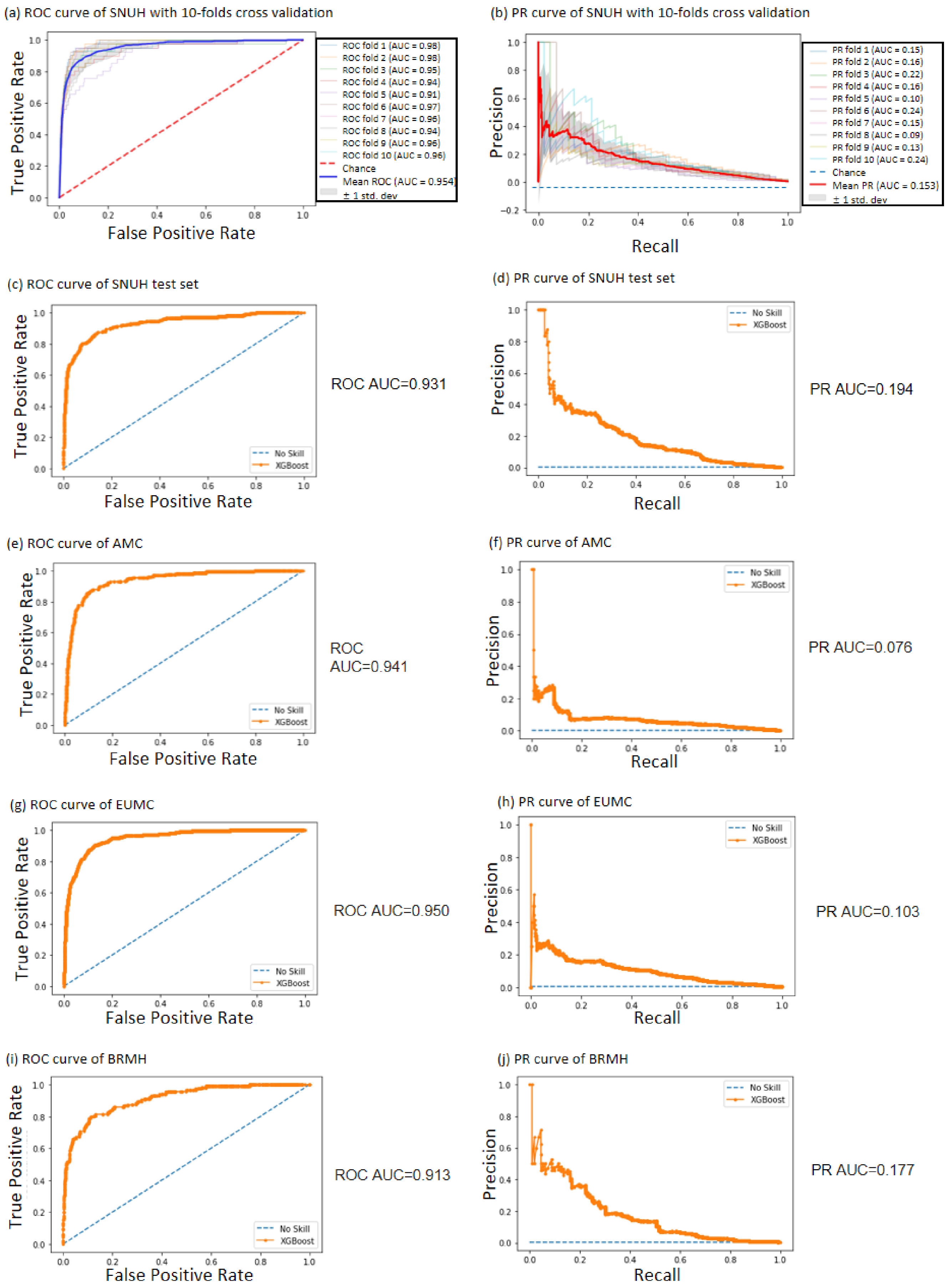

ROC, receiver operating characteristic; PR, precision-recall; AUC, area under the curve; SNUH, Seoul national university hospital; AMC, Asan medical center; EUMC, Ewha womans university hospital; BRMH, Boramae hospital.

Supplementary Figure 5. Calibration curve of XGBoost model of SNUH with 10-fold cross validation on the SNUH test set, AMC, EUMC, and BRMH datasets.

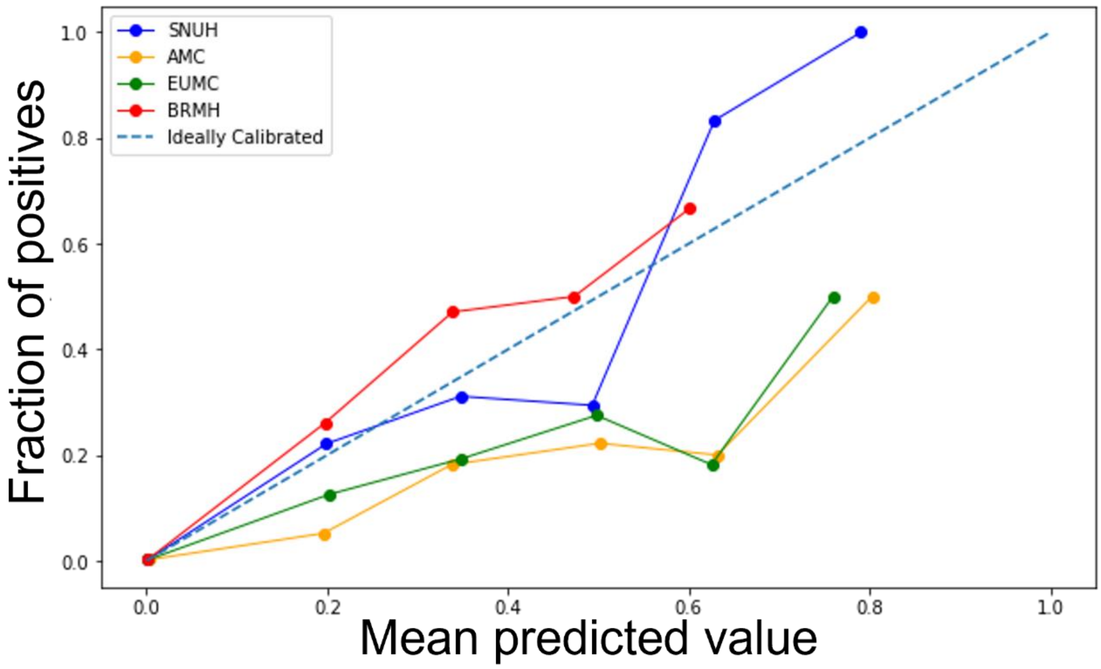

SNUH, Seoul national university hospital; AMC, Asan medical center; EUMC, Ewha womans university hospital; BRMH, Boramae hospital.
